# Functional Categories Associated with Clusters of Genes That Are Co-Expressed across the NCI-60 Cancer Cell Lines

**DOI:** 10.1371/journal.pone.0030317

**Published:** 2012-01-24

**Authors:** Barry R. Zeeberg, William Reinhold, René Snajder, Gerhard G. Thallinger, John N. Weinstein, Kurt W. Kohn, Yves Pommier

**Affiliations:** 1 Laboratory of Molecular Pharmacology, Center for Cancer Research, National Cancer Institute, National Institutes of Health (NIH), Bethesda, Maryland, United States of America; 2 Institute for Genomics and Bioinformatics, Graz University of Technology, Graz, Austria; University of Chicago, United States of America

## Abstract

**Background:**

The NCI-60 is a panel of 60 diverse human cancer cell lines used by the U.S. National Cancer Institute to screen compounds for anticancer activity. In the current study, gene expression levels from five platforms were integrated to yield a single composite transcriptome profile. The comprehensive and reliable nature of that dataset allows us to study gene co-expression across cancer cell lines.

**Methodology/Principal Findings:**

Hierarchical clustering revealed numerous clusters of genes in which the genes co-vary across the NCI-60. To determine functional categorization associated with each cluster, we used the Gene Ontology (GO) Consortium database and the GoMiner tool. GO maps genes to hierarchically-organized biological process categories. GoMiner can leverage GO to perform ontological analyses of gene expression studies, generating a list of significant functional categories.

**Conclusions/Significance:**

GoMiner analysis revealed many clusters of coregulated genes that are associated with functional groupings of GO biological process categories. Notably, those categories arising from coherent co-expression groupings reflect cancer-related themes such as adhesion, cell migration, RNA splicing, immune response and signal transduction. Thus, these clusters demonstrate transcriptional coregulation of functionally-related genes.

## Introduction

The NCI-60 is a panel of 60 human cancer cell lines that has been used by the Developmental Therapeutics Program (DTP) of the U.S. National Cancer Institute to screen compounds plus natural products since 1990 [1], [2]. The NCI-60 panel includes cell lines from colorectal (CO), renal (RE), ovarian (OV), prostate (PR), lung (LC), breast (BR), and central nervous system (CNS) cancer origin, as well as leukemias (LE) and melanomas (ME). We and our many collaborators around the world have profiled the NCI-60 more comprehensively at the DNA, RNA, protein, mutation, functional, and pharmacological levels than any other set of cells in existence [1], [2], [3], [4], [5], [6]. The NCI-60 data have been widely used in cancer research and bioinformatics, but the multiple datasets may be most informative for the recognition of complex ‘biosignatures.’ Such biosignatures may in turn lead to increased understanding of cell phenotypes and pathway relationships within the cell.

We previously developed GoMiner [7] and High-Throughput GoMiner [8], applications that organize lists of “interesting” genes (for example, under- and over-expressed genes from a microarray experiment) for biological interpretation in the context of the Gene Ontology [9], [10]. GoMiner and related tools typically generate a list of significant functional categories. In addition to lists and tables, High-Throughput GoMiner can provide two kinds of clustered image maps (CIMs) as graphical output. Integrative *categories versus experiments* CIMs capture the relationships between categories and multiple experiments; individual *categories versus genes* CIMs capture the relationships between categories and genes. Both types of CIMs are used to present the results in the present work.

In the past decade, systems biology has become increasingly prominent as the numbers of analyzable genes and biological parameters have increased, and is beginning to show their functional relationships. A standard approach for studying systems biology with genomic data is to cluster genes whose expression profiles co-vary either over a time course or across multiple samples. For example, Garraway *et al.* [11] performed an integrated supervised analysis of SNP array and gene expression data to identify MITF as a lineage survival oncogene amplified in malignant melanoma. A number of additional gene expression microarray demonstrate the potential of gene co-expression studies. For example, Prieto *et al.* [12] used the Affymetrix HGU133A platform to identify co-expression networks in a diversity of human tissue samples. Their network revealed a map of coexpression clusters organized in well-defined functional constellations. Two major regions in this network corresponded to genes involved in nuclear and mitochondrial metabolism. That study is not directly relevant to cancer, though, since no cancer tissues were included in the study. Choi *et al.* [13] did study cancer tissues, but had unfortunately culled published data from what would now be considered to be outdated (Affymetrix U95A) or unreliable (cDNA) platforms. Also, the data obtained on different platforms needed to be reconciled, and the date of the studies preceded the availability of reliable resources like AffyProbeMiner [14] and SpliceCenter [15]. Nevertheless, Choi was able to detect functional differences between normal growth and cancer in terms of gene coexpression changes in broad areas of physiology: energy metabolism, the cell cycle, immune activation and collagen production.

Other studies have been focused on tissue-specific genes. Cho *et al.* [16] revealed many pathways related to the pathophysiology of lung cancer: Cytokine Network and TNF/Stress Related Signaling pathway pair; thrombin signaling and protease-activated receptors pathway; Cell Cycle: G1/S Check Point and Inhibition of Cellular Proliferation by Gleevec. Likewise, the studies of Lai *et al.* [17] were restricted to prostate cancer and developed a statistical method for identifying differential gene–gene co-expression patterns in different cell states. For a gene of interest, other genes are selected that have differential gene–gene co-expression patterns with this gene in different cell states. By using the tumor suppressor genes TP53, PTEN and RB1 as the gene of interest, selected genes included hepsin, GSTP1 and AMACR.

The present study was undertaken to test the hypothesis that genes from similar functional categories tend to exhibit comparable patterns of expression across cell lines from a broad tissue of origin spectrum (*i.e*, the NCI-60 cell lines). This hypothesis was generated in the course of our recent study showing that the nuclear-encoded mitochondrial genes are coregulated among each other and with the MYC gene across the NCI-60 [18], [19]. The present analysis was performed with the enhanced expression data in CellMiner (http://discover.nci.nih.gov/cellminer) [20], [21]. Those data are of superior quality, since they are obtained by compilation of five microarray platforms (see details in Method section). They also address the generality of the coregulation processes since the NCI-60 comprises a particularly rich set of samples from 9 tissue types with high reproducibility.

## Results and Discussion

### Global overview of the strategy and process flow

**Figure 1:**
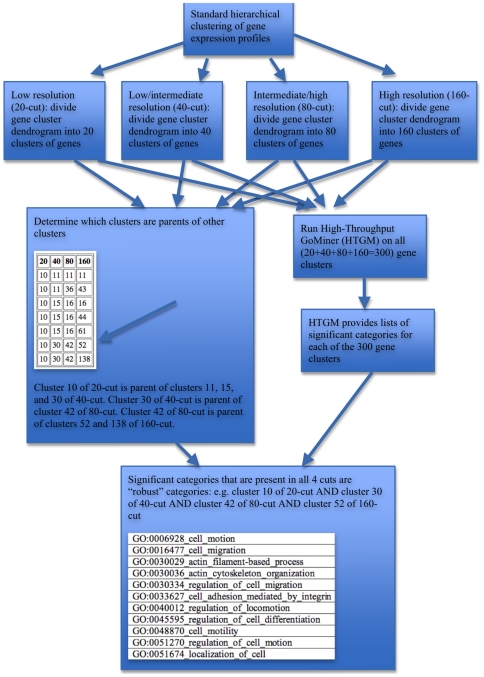
Flow diagram illustrating the process of determining robust functional categories of coregulated genes across the NCI-60.

A flow diagram (Figure 1) provides a global overview of the process flow. We first performed standard hierarchical clustering on the gene expression profiles across the NCI-60 cell lines. We then cut the resulting cluster tree to achieve 4 levels of cuts, requesting (from lowest to highest resolution) 20, 40, 80, or 160 gene clusters (resulting in a total of 20+40+80+160 = 300 gene clusters). This scheme generated families of clusters such that a cluster of the 20-cut was a parent of a child cluster in the 40-cut, and so on. A cluster of the 20-cut may have one or more such children, but each child has only one parent. Thus, each cluster family could be uniquely designated by the cluster number of its 160-cut. The gene sets for each of the 300 clusters were submitted to High-Throughput GoMiner (HTGM) to determine the significant Gene Ontology Consortium (GO) categories associated with each gene set. The GO categories that were present across all 4 cuts of a cluster family were deemed to be *robust* categories associated with that family. The significance of robustness is that a robust category is independent of the particular degree of resolution used for cutting the gene cluster tree. Thus, the robust categories are more focused and reliable than non-robust categories that are significant for some particular cut, but not for all cuts.

### Gene clustering based on co-expression

Using this strategy and processing flow, we set out to examine the whole dataset for the 16,821 genes in CellMiner with high quality expression data across the multiple mRNA expression platforms in the NCI-60 cell lines. Hierarchical clustering of the gene expression profiles was explored at 4 levels of resolution by requesting cuts containing 20-, 40-, 80-, or 160-clusters.

### GO categories associated with each co-expression cluster

**Figure 2:**
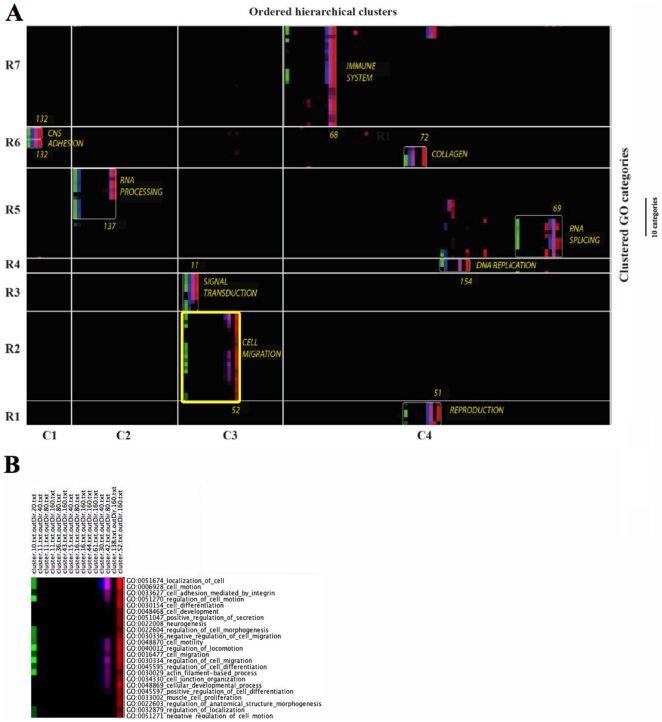
GO categories *versus* ordered hierarchical clusters CIM. (**A**) Compact version. The full version is available as Figures S1A, B. Only categories with FDR<0.10 for at least one cut are represented. The coordinates of the clusters (*e.g.*, R1, C1) are shown in Table 1. The HTGM FDR for the GO categories for the 20-, 40-, 80-, and 160-cuts are given in green, blue, pink, and red, respectively. A bright shade corresponds to high correlation (i.e. a low FDR), and a darker shade corresponds to an FDR close to the threshold of 0.10. The cluster numbers for the 160-cuts are shown at the right of each encircled grouping. (B) Blowup of the cluster 52 family grouping derived from Figure 2A.

We ran High-Throughput GoMiner (HTGM) on the gene sets in all 300 clusters, and asked whether there would be any GO categories present across all 4 levels of cuts of a cluster family. That result was best visualized by a novel type of “categories *versus* experiments” CIM (Figures 2A, S1A,B). Only the rows were clustered, since the columns had already been pre-arranged in a special sort order: starting with one of the clusters from the 20-cut, we linked that cluster with the cluster(s) of the 40-cut that are the “children” of the 20-cut. That process was applied recursively to all 4 cuts. To facilitate visualization of the cuts, we took advantage of a new feature of the Genesis clustering program to assign a distinct color scale to each cut. We outlined the same groups of categories that were statistically significant and that had mutually-related biological functionality within the NCI-60 clusters (white rectangles in Figures 2A and S1B). The cluster family numbers and functional designations appear adjacent to each encircled group. At the right of Figure 2A is a scale indicator showing the height occupied by 10 rows of categories. The coordinates of clusters in Figure 2 are given in Table 1, and the robust categories depicted in Figure 2A are given in Table S1.

**Table 1:** Coordinates of clusters in Figures 2A and S1A, B.

Generalized functionality	Cluster number within the 160-cut	Row	Column
immune system	68	7	4
CNS	132	6	1
adhesion	132	6	1
collagen	72	6	4
RNA processing	137	5	2
RNA splicing	69	5	4
DNA replication	154	4	4
signal transduction	11	3	3
cell migration	52	2	3
reproduction	81	1	4

Rows and columns were divided so as to result in a single cluster family *per* area, when possible.

Figure 2A clearly shows well-defined cluster families that arise from the convergence of coherent gene expression and coherent biological processes with an overriding GO category. That convergence is especially clear for several cluster families (the cluster number for the 160-cut component of the family is given in parentheses): cell migration (52), signal transduction (11), reproduction (51), cell adhesion (132), collagen (72), immune system (68), RNA processing (137), RNA splicing (69) and DNA replication (154). Thus, each cluster was defined by a specific gene expression profile and a specific and unifying GO categorization.

We were gratified to find that we could identify 64 robust categories (Table S1), comprising 15 generalized GO functionalities, all of which (with the exception of eye pigmentation) are closely related to cancer. To better illustrate the operational definition and concept of robustness, we have constructed a blowup (Figure 2B) of the cluster 52 family grouping outlined in yellow in Figure 2A. The cluster 52 family grouping consists of the descendants of cluster 10 of the 20-cut, as tabulated in the panel “Determine which clusters are parents of other clusters” in the flow diagram (Figure 1). That panel shows that the path to cluster 52 of the 160-cut includes cluster 30 of the 40-cut and cluster 42 of the 80-cut. In Figure 2B, note that 4 different color scales differentiate the 4 cuts (*e.g.*, green, blue, lavender, and red designate 20-, 40-, 80-, and 160-cuts, respectively). For example, HTGM analysis showed that GO:0051674_localization_of_cell was statistically significant in clusters 10, 30, 42, and 52 of the 20-, 40-, 80-, and 160-cuts, respectively. Thus, GO:0051674_localization_of_cell was designated as being a robust category. In contrast, GO:0048468_cell_development was significant only in cluster 52 of the 160-cut, and was thus not designated as robust. Note that the panel in the flow diagram shows 7 family groupings derived from cluster 10 of the 20-cut. The present figure shows that none of the family groupings other than 10/30/42/52 contains a robust category, although some contain significant categories (*e.g.*, 10/11/36/43 contains GO:0051674_localization_of_cell as a significant but not robust category).

The robust categories for the cluster family corresponding to cluster 52 of the 160-cut are listed in the bottom panel of the flow diagram in Figure 1. Those robust categories focus on cell migration, whereas the (robust plus non-robust) significant categories are more diverse, generally reflecting neuron development, immune response, and epithelial-mesenchymal transition (EMT) in addition to cell migration (see “Categories *versus* genes” CIMs below).

### Public database to allow exploration of the results in Figure 2A

**Figure 3:**
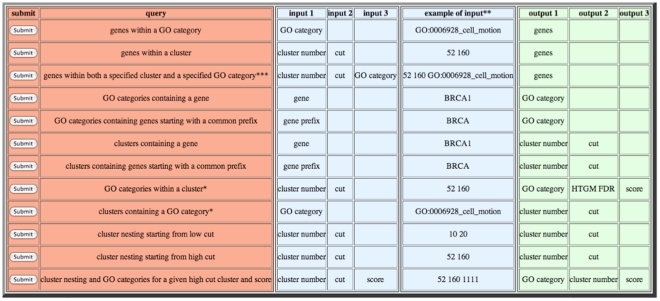
Screenshot of the front end of the database consisting of a convenient table of clickable queries and examples of the corresponding input and output parameters.

To facilitate future research using the clustering and functional categorization results reported here, we provide a public database. Several pre-constructed MySQL queries can be issued to retrieve information from a database containing the results in Figure 2A and its expanded version Figure S1B. A typical query might involve retrieving the list of genes within a specified cluster that map to a specified GO category. A graphical user interface (GUI) for issuing the desired query is provided at URL http://discover.nci.nih.gov/NCI60/menu.table.html. The URL contains a convenient table of clickable queries and examples of the corresponding input and output parameters (Figure 3). A PowerPoint tutorial for using the database is available from Supplementary Materials (Powerpoint S1).

### “Categories *versus* genes” CIMs

**Figure 4:**
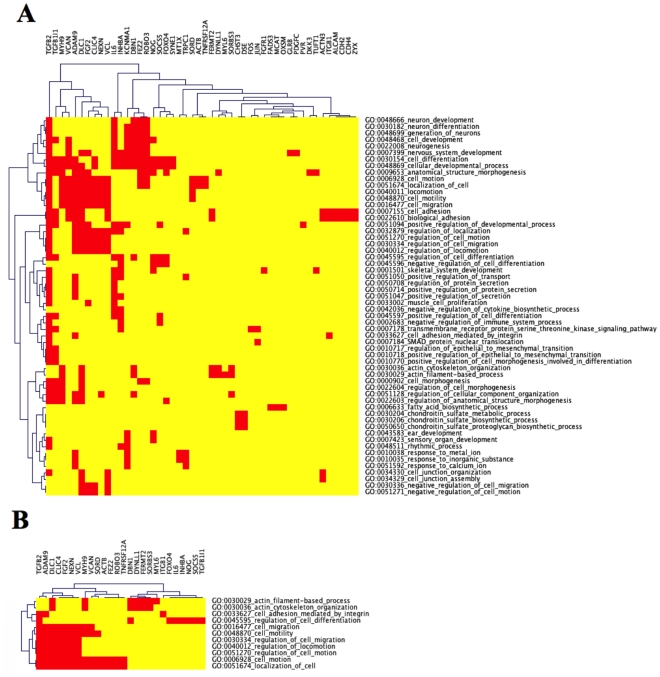
Categories *versus* genes CIMs for the significant categories in cluster 52 of the 160-cut and the 48 genes of Step 4 in **Table 2** (A), and for the robust categories of the cluster family containing cluster 52 of the 160-cut and the 26 genes of Step 5 in **Table 2** (B).

To illustrate one type of biological information that can be gleaned from the clustering strategy that we used, we delineate the relationship between genes and functional categories for cluster 52 of the 160-cut, by constructing a “categories *versus* genes” CIM for the significant categories (Figure 4A) and for the robust categories (Figure 4B). Further details are presented in the Method.

The significant categories CIM is a superset of the robust categories CIM with respect to both genes and categories. As mentioned above, the robust categories focus strongly on cell migration, whereas the significant categories of cluster 52 of the 160-cut are more diverse, generally reflecting neuron development, immune response, and EMT in addition to cell migration. The statistics for the two CIMs are summarized in Step numbers 4 and 5 in Table 2.

**Table 2:** Number of genes and categories surviving each processing step for cluster 52 of the 160-cut.

Step number	Process	Corresponding step in Figure 1	Number of genes	Fraction of CellMiner	Number of GO categories
1	Total set of human genes represented in CellMiner	NA	16,821	1.00000	NA
2	Genes having HGNC symbol and mapping to the Biological Process branch of GO	“Standard hierarchical clustering…”	6,477	0.38505	NA
3	Genes in cluster 52 of the 160-cut	“High resolution (160-cut)…”	83	0.00493	NA
4	Genes in cluster 52 of the 160-cut mapping to a (statistically) significant GO category	“HTGM provides lists…”	48	0.00285	58
5	Genes in cluster 52 of the 160-cut mapping to a robust GO category	“Significant categories that are present in all 4 cuts…”	26	0.00154	11

For the robust categories CIM (Figure 4B), in some instances there is substantial overlap between the genes in categories, such as occurs for the bottom 7 categories (the “cell migration” group) in the CIM. In this situation, we interpret those categories as being largely redundant with respect to one another. A more informative situation occurs when there is not complete redundancy, but rather when there is only partial overlap between (groups of) categories, such as the above-mentioned cell migration group, and the top four categories in the CIM. Such partial overlap may reveal “cross-talk” among various biological functionalities. The category relationships may reflect the participation of cell migration components, such as cytoskeleton and integrins.

For the significant categories (Figure 4A), TGFB2 mediates cross-talk between the neuron differentiation and the cell migration groups of categories. More striking is the separation of the bulk of the cell migration-related (*i.e.*, TGFB1I1, MYH9, VCAM, ADAM9, DLC1, FGF2, CLIC4, NEXN, and VCL) and neuron-related genes (*i.e.*, IL6, INHBA, KCNMA1, DBN1, FEZ2, ROBO3, and NOG). Thus, for the most part, different sets of genes correlate with those 2 functionalities, and the reason for their appearing in the same cluster family 52 of the 160-cut (by virtue of highly correlated gene expression profiles) indicates an intimate relationship between cell migration and neuron development that requires future investigation.

### Conclusions

The comprehensive nature of the NCI-60 gene expression dataset, together with the broad range of tissue of origin represented, allowed us to gain insight into the systems biology of cancer cells by identifying multiple clusters of genes that co-vary across the 60 cell lines.

To further characterize the genes within each cluster, we used the Gene Ontology (GO) Consortium database in conjunction with the GoMiner tool to determined functional associations. GoMiner analysis revealed that the genes in many clusters are associated with coherent GO biological process categories, such as cell migration, signal transduction, reproduction, cell adhesion, collagen, immune system, RNA processing, RNA splicing, and DNA replication.

The novel features of our approach are (1) co-expression analysis of the high-quality gene expression profiles afforded by the recently-available composite transcriptome profile based on the integrated gene expression levels from five platforms, (2) the use of GO categorization to find robust categories that do not depend on choosing a particular level of resolution for cutting the cluster dendrogram, and (3) using the genes in selected clusters to generate future research directions, such as the cell migration genes in cluster 52 of the 160-cut (Kohn *et al.*, manuscript in preparation). To our knowledge, none of these features have been studied/implemented previously.

One type of new insight is the elucidation of novel gene connections based on the dual criteria of co-expression and co-ordinated functional categorization. This connection can be visualized by examining the genes in those GO categories having partial overlap using the gene *versus* categories type of HTGM CIM (see for example TGFB2 cross-talk between the neuron differentiation and the cell migration categories in Figure 4A).

A second type of new insight is the elucidation of the most highly co-regulated pathways, with confirmation by related functional categorization of the genes in the pathway. For example, many of the genes in cluster 52 of the 160-cut are involved in a highly coordinated cell migration pathway (Kohn *et al.*, manuscript in preparation).

## Materials and Methods

### CellMiner

#### NCI-60 transcript expression

Gene transcript expression was determined using probes from five platforms. These include, from Affymetrix (Affymetrix Inc., Sunnyvale, CA), the ∼60,000 feature Human Genome U95 Set (HG-U95) [5], the ∼44,000 feature Human Genome U133 array (HG-U133) [5], the ∼47,000 feature Human Genome U133 Plus 2.0 Arrays (HG-U133 Plus 2.0); and the ∼5,500,000 feature GeneChip Human Exon 1.0 ST array (GH Exon 1.0 ST) [19]. Also included from Agilent (Agilent Technologies, Inc., Santa Clara, CA) was the ∼41,000 feature Whole Human Genome Oligo Microarray [3]. All Affymetrix platforms were normalized by Guanine Cytosine Robust Multi-array Analysis, or GCRMA [22]. Agilent mRNA probes were normalized based on their detection in at least 10% of the cell lines, using GeneSpring GX by i) setting any gProcessedSignal value less than 5 to 5, ii) transforming the gProcessedSignal or gTotalGeneSignal to Logbase 2, and iii) normalizing per array to the 75^th^ percentile [3]. Our relational database, CellMiner, at <http://discover.nci.nih.gov>, can be used to access data from the HG-U95, HG-U133, HG-U133 Plus 2.0 and Agilent Whole Human Genome Oligo Microarrays.

Probes (Agilent) or probe sets (Affymetrix) were then passed through the following quality control criteria prior to their use in determining relative gene expression levels. First, average probe set intensity ranges (meant to include Agilent probes in the following text) were determined. Probe sets with an intensity ranges<or equal to 1.2 log_2_ were dropped. The probe sets number for a gene that passed this criteria was determined, and 25% of that number calculated. Pearson's correlations were determined for all possible combinations of the remaining probe sets (for each gene). Each probe set's average correlation was determined as compared to all others (for a single gene). Next, those probe sets with average correlations of less than 0.30 were removed. Following this step, probe sets with the lowest average correlations <0.60 were dropped. The remaining probe set/probe set correlations combinations were then recalculated. The lowest average correlation probe set continued to be dropped, and the average recalculated until either all average correlations were≥to 0.60, or until we reached the 25% level of the original probe set number (calculated above).

These procedures yielded accurate transcript intensity values that were highly reproducible and internally consistent. Additionally contributing to the high quality of the data, we think, were the following: (1) Cell growth, harvesting and quality control were done primarily by one person (W. Reinhold). (2) Quality control of individual probe sets were based on a minimum intensity range of <1.2 log2 and pattern correlation of >0.60. This provides protection against sporadically bad probe sets. (3) Transformation of the data into z scores [23] by subtraction of the 60 cell line means and division by the standard deviations provided protection against single-platform anomalies, and allowed comparison of all probe set data. Z scores averages were determined for all available (18,412) genes for each cell line. Details of the z-score computation are provided in the Supplemetary Materials (Document S1). These calculations were done in Java.

Each step in the process of extracting genes from CellMiner [21], and selecting those that match both HUGO Gene Nomenclature Committee symbols (HGNC) [24] symbol as well as a GO database annotation, results in a “loss” of genes. The degree of loss in each step is summarized in Table S2. For instance, 29,017 and 16,821 genes are represented in HGNC and the five-platform transcript expression analysis, respectively. The subset of genes represented in HGNC is 11,767/16,821 = 69.9%. That figure is higher than the overall percentage of approximately 55% of all human genes that are represented by HGNC (Zeeberg *et al.*, unpublished). The subset of HGNC genes represented in the Biological Process ontology of GO (under the conditions specified in Table S2) comprises a somewhat disappointing 7,654/29,017 = 26.4%. The overall yield of five-platform genes that have both HGNC and GO Biological Process annotations is 6,477/11,767 = 55.0%.

### Downloading and pre-processing genes from CellMiner

A special request was made to the system administrator for the complete set of gene expression profiles. That download would have been too large to perform through the standard web interface. The values for each gene were based on a consensus of five microarray platforms, and are expressed as Z-scores, as detailed in the Supplementary Materials and as described previously [19].

The data were pre-processed by pre-selecting only those genes that have both an HGNC symbol and annotation in the GO Biological Process ontology. Each gene profile vector was scaled to zero mean and unit variance.

### Gene clustering based on co-expression

An R language (http://www.R-project.org) [25] script was developed to perform hierarchical clustering of the gene expression profiles across the NCI-60. Since genes may function positively or negatively within a network, we wanted genes that were highly correlated and highly anti-correlated to be assigned to the same cluster, so we specified a distance metric of 1-abs(cor(t(mat)))/2. We also specified complete linkage clustering.

We used the R function *cutree()* to cut the resulting hierarchical cluster tree into 20, 40, 80, and 160 clusters. Those clusters had two important properties:

- The total set of genes in the cluster tree was divided (completely and without duplication) among the clusters. That is, each gene in the original set appeared in exactly one cluster.
- The clusters of the 40-cut were nested within the clusters of the 20-cut. That is, each cluster of the 40-cut was a subset of a single cluster of the 20-cut. That pattern was maintained recursively through all levels of cuts.

The gross distribution of genes for all 300 (*i.e.*, 20+40+80+160) clusters is shown in Table S3. Each cluster was subsequently analyzed by GoMiner (see next section). We performed multiple cuts because we wanted to prioritize those GO categories that were independent of the particular cutting pattern (see the Methods section “Scoring GO categories”).

The relationship between the clusters in successive cuts (e.g., 20 and 40, 40 and 80, or 80 and 160) was delineated by a table generated by the sequence of R calls exemplified for 20 and 40 as:
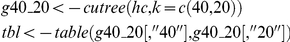
The resulting table showed which cluster(s) in the 40-cut arose from each cluster in the 20-cut. *Cluster families* could be defined by starting with one of the clusters in the 20-cut, and using the 20- and 40-cut table to determine all of the 40-cut clusters that were derived from that 20-cut cluster. That process was repeated in turn for those 40-cut clusters by using the 40- and 80-cut table, and so forth. The set of the selected 20-cut cluster plus a single derived cluster from each of the 40-, 80-, and 160-cuts constituted a cluster family.

### High-Throughput GoMiner (HTGM)

GoMiner [7] is a tool for biological interpretation of ‘omic’ data, including data from gene expression microarrays and state of the art sequencing technologies. It leverages the Gene Ontology (GO) to identify “biological processes,” “molecular functions,” and “cellular components” represented in a list of genes. High-Throughput GoMiner (HTGM) [8], which was used for many of the analyses reported here, is an enhancement of GoMiner that efficiently performs the computationally-challenging task of automated batch processing of an arbitrary number of such gene lists.

A GO category is *enriched* if the number of changed genes that HTGM assigned to it is statistically significantly greater than the number expected by chance. A category is considered *significant* if its Fisher's Exact p-value and its false discovery rate (FDR) are both less than or equal to a user-selected threshold (typically 0.10; on rare occasion, the p-value can exceed the threshold although the FDR is below the threshold, and we usually want to reject such instances). See [7], [8] for detailed discussions of GoMiner and HTGM, including calculations of statistical significance.

We ran all clusters derived from the cuts for 20-, 40-, 80-, and 160-cut clusters, a total of 300 input files, in a single HTGM run. The parameters used in all of the HTGM analyses are listed in Table S4.

The average genes/cluster at the 160-cut level was approximately 40, which we would usually consider to be too few genes to submit to GoMiner. However, in this instance, as shown below, we do find many significant and functionally consistent GO clusters. Thus, the prior hierarchical clustering of the genes based on expression appears to have pre-focused the genes in a functionally coherent manner so as to compensate for the low statistical power of a small set.

The gross distribution of GO categories that results from running GoMiner on the 300 clusters comprising the 20-, 40-, 80-, and 160-cuts is shown in Table S5. Thus, similarity of gene expression profiles sometimes, but not always, implies coherence of biological function. The fraction of clusters with at least one significant category decreased modestly from 0.55 (for the 20-cut) to 0.41 (for the 160-cut).

### Sorting clusters within cluster families

Cluster families are defined in the Methods section “Gene profile-based hierarchical clustering.” We devised an algorithm for sorting the clusters within a cluster family for eventual display as a CIM image. The algorithm uses tables generated by R code (see “Gene profile-based hierarchical clustering”) to provide the proper global ordering of clusters derived from one another in different cuts for 20-, 40-, 80-, and 160-cut clusters. Briefly, a cluster family consists of a given 20-cut, and the 40-cut(s) derived from that 20-cut, and so forth.

### Scoring GO categories

Each GO category that was significant in at least one hierarchical cluster was scored according to its presence in clusters of each of the 20-cut families. The score was represented as a bit string exemplified, for example, as 1101, which indicates that the category was present in a cluster derived from the 160-, 80-, and 20-cut, but not in any cluster from the 40-cut. The score of the category was taken as the maximal score over all 20-cut families [There are, by definition, twenty 20-cut families. A given category will have a score in each of the 20-cut families (*i.e.*, twenty scores). Most of those scores will be 0000]. The distribution of scores and a listing of the categories according to score are given in Tables S6 and S7, respectively. The categories with a score of 1111 were prioritized and designated as “robust” categories, as they were not dependent on the particular type of cut that was used.

### Clustered image maps

Clustered image maps (CIMs), first introduced for omic studies in the mid-1990's by members of our group [6], were produced here with the Genesis program [26]. We selected the Euclidean distance metric and average linkage for hierarchal clustering. To facilitate visualization, we implemented a recently-added optional feature of GoMiner to remove very large generic categories from all CIMs.

#### Integrative “categories versus experiments” CIM

A new feature of Genesis allows each column of the CIM to be assigned one of six available color scales. Columns that are conceptually related can all be assigned the same color scale, and thus easily recognized visually after clustering. We used that feature to identify columns that arose from the same instance of cutting the hierarchical cluster tree. For instance, as described in more detail in the Methods section “hierarchical clustering,” a hierarchical cluster tree was cut into 20, 40, 80, and 160 clusters. All clusters of the 20-cut were designated with green color scale, and the others were designated as dark blue, lavender, and red, respectively.

#### Individual “categories versus genes” CIM

To better delineate the relationship between genes and functional categorization, we constructed a “categories *versus* genes” CIM for those categories having a score of 1111. The genes in the CIM are taken from clusters that simultaneously met both of the following two criteria:

- the clusters belong to the cluster family in which the category achieves the score of 1111
- the category is a member of the cluster

Those restrictions take into account the following types of situations:

- a category might be found in clusters belonging to two different cluster families, but we do not want to contaminate the CIM with genes that are associated with the category in the suboptimal cluster family
- within the optimal cluster family, there are generally many clusters that do not contain the category of interest, and we do not want to contaminate the CIM with genes that are associated with the clusters that do not include the category of interest, even though the cluster is a member of the optimal cluster family

## Supporting Information

Figure S1 CIM of categories versus cluster groups. (A) Each cluster group consists of a set of related clusters from the 20-, 40-, 80-, and 160-cut. The clusters are related by successive splittings from the 20-cut cluster. Clusters from a given cut are representd by a distinct color scale, as shown on the top of the figure. Robust categories and functionally-related adjacent regions are designated within a white rectangle. (B) Compacted version of CIM of categories categories versus cluster groups. Only those rows and columns containing robust categories were retained. The cluster number for the 160-cut is shown at the right of each encircled grouping.
(PDF)

Table S1 Robust GO categories for all cluster families.
(DOC)

Table S2 Loss of genes in each step of the process of extracting genes from CellMiner and selecting those that match both an HGNC symbol and a GO database annotation.
(DOC)

Table S3 The gross distribution of genes submitted to GoMiner for all 300 clusters.
(DOC)

Table S4 Parameters used in HTGM analyses.
(DOC)

Table S5 The gross distribution of GO categories resulting from running GoMiner on all 300 clusters.
(PDF)

Table S6 Distribution of scores.
(DOC)

Table S7 Categories according to score.
(DOC)

Powerpoint S1 Database tutorial.
(PPT)

Document S1 Z-scores.
(JPG)
