# Supplementary material for: Functional Categories Associated with Clusters of Genes That Are Co-Expressed across the NCI-60 Cancer Cell Lines
Source: PLoS One. 2012 Jan 24;7(1):e30317. doi: 10.1371/journal.pone.0030317 (PMC3265467; doi:10.1371/journal.pone.0030317)
Supplement: Table S1 — Robust GO categories for all cluster families. (DOC) [file pone.0030317.s002.doc]

**Table S1. Robust GO categories for all cluster families**

| **Cluster number within the 160-cut*** | **Generalized GO functionality** | **Number of robust GO categories**** | **GO category** |
| --- | --- | --- | --- |
| 8 | translational elongation | 1 | GO:0006414_translational_elongation |
| 11 | signal transduction | 6 | GO:0007265_Ras_protein_signal_transduction |
|  |  |  | GO:0009966_regulation_of_signal_transduction |
|  |  |  | GO:0010646_regulation_of_cell_communication |
|  |  |  | GO:0035023_regulation_of_Rho_protein_signal_transduction |
|  |  |  | GO:0046578_regulation_of_Ras_protein_signal_transduction |
|  |  |  | GO:0051056_regulation_of_small_GTPase_mediated_signal_transduction |
| 32 | mRNA metabolic process | 5 | GO:0000375_RNA_splicing__via_transesterification_reactions |
|  |  |  | GO:0000377_RNA_splicing__via_transesterification_reactions_with_bulged_adenosine_as_nucleophile |
|  |  |  | GO:0000398_nuclear_mRNA_splicing__via_spliceosome |
|  |  |  | GO:0006397_mRNA_processing |
|  |  |  | GO:0008380_RNA_splicing |
| 52 | cell migration | 11 | GO:0006928_cell_motion |
|  |  |  | GO:0016477_cell_migration |
|  |  |  | GO:0030029_actin_filament-based_process |
|  |  |  | GO:0030036_actin_cytoskeleton_organization |
|  |  |  | GO:0030334_regulation_of_cell_migration |
|  |  |  | GO:0033627_cell_adhesion_mediated_by_integrin |
|  |  |  | GO:0040012_regulation_of_locomotion |
|  |  |  | GO:0045595_regulation_of_cell_differentiation |
|  |  |  | GO:0048870_cell_motility |
|  |  |  | GO:0051270_regulation_of_cell_motion |
|  |  |  | GO:0051674_localization_of_cell |
| 68 | immune system | 8 | GO:0001775_cell_activation |
|  |  |  | GO:0002376_immune_system_process |
|  |  |  | GO:0002682_regulation_of_immune_system_process |
|  |  |  | GO:0006955_immune_response |
|  |  |  | GO:0042110_T_cell_activation |
|  |  |  | GO:0045058_T_cell_selection |
|  |  |  | GO:0045321_leukocyte_activation |
|  |  |  | GO:0046649_lymphocyte_activation |
| 69 | RNA splicing | 4 | GO:0000375_RNA_splicing__via_transesterification_reactions |
|  |  |  | GO:0000377_RNA_splicing__via_transesterification_reactions_with_bulged_  adenosine_as_nucleophile |
|  |  |  | GO:0000398_nuclear_mRNA_splicing__via_spliceosome |
|  |  |  | GO:0022618_ribonucleoprotein_complex_assembly |
| 72 | collagen | 3 | GO:0030198_extracellular_matrix_organization |
|  |  |  | GO:0030199_collagen_fibril_organization |
|  |  |  | GO:0043588_skin_development |
| 81 | reproduction | 3 | GO:0007565_female_pregnancy |
|  |  |  | GO:0045086_positive_regulation_of_interleukin-2_biosynthetic_process |
|  |  |  | GO:0051704_multi-organism_process |
| 83 | eye pigmentation | 1 | GO:0048069_eye_pigmentation |
| 95 | RNA splicing | 4 | GO:0000245_spliceosome_assembly |
|  |  |  | GO:0006376_mRNA_splice_site_selection |
|  |  |  | GO:0006397_mRNA_processing |
|  |  |  | GO:0008380_RNA_splicing |
| 114 | ribonucleoprotein complex biogenesis | 2 | GO:0006396_RNA_processing |
|  |  |  | GO:0022613_ribonucleoprotein_complex_biogenesis |
| 132 | adhesion, CNS | 5 | GO:0007399_nervous_system_development |
|  |  |  | GO:0007416_synaptogenesis |
|  |  |  | GO:0016337_cell-cell_adhesion |
|  |  |  | GO:0016339_calcium-dependent_cell-cell_adhesion |
|  |  |  | GO:0050808_synapse_organization |
| 137 | RNA processing | 8 | GO:0006302_double-strand_break_repair |
|  |  |  | GO:0006378_mRNA_polyadenylation |
|  |  |  | GO:0016071_mRNA_metabolic_process |
|  |  |  | GO:0016567_protein_ubiquitination |
|  |  |  | GO:0031124_mRNA_3'-end_processing |
|  |  |  | GO:0032446_protein_modification_by_small_protein_conjugation |
|  |  |  | GO:0043631_RNA_polyadenylation |
|  |  |  | GO:0070647_protein_modification_by_small_protein_conjugation_or_  removal |
| 140 | reproduction | 1 | GO:0006959_humoral_immune_response |
| 154 | DNA replication | 2 | GO:0006259_DNA_metabolic_process |
|  |  |  | GO:0006260_DNA_replication |

The relationship of these categories and clusters can be viewed graphically in the CIMs in Figures 2, S1A, B.

*The cluster family is designated by the cluster number of the 160-cut.

**The total number of robust categories is 64.
