# Supplementary material for: Functional Categories Associated with Clusters of Genes That Are Co-Expressed across the NCI-60 Cancer Cell Lines
Source: PLoS One. 2012 Jan 24;7(1):e30317. doi: 10.1371/journal.pone.0030317 (PMC3265467; doi:10.1371/journal.pone.0030317)
Supplement: Table S2 — Loss of genes in each step of the process of extracting genes from CellMiner and selecting those that match both an HGNC symbol and a GO database annotation. (DOC) [file pone.0030317.s003.doc]

Table S2. Loss of genes in each step of the process of extracting genes from CellMiner and selecting those that match both an HGNC symbol and a GO database annotation

| **gene set** | **∩HGNC** | **∩CM** | **∩HGNC∩BP** |
| --- | --- | --- | --- |
| **HGNC** | 29017 | 11767 | 7654 |
| **CM** | 11767 | 16821 | 6477 |

CM and BP are abbreciations for CellMiner and GO biological process ontology, respectively.

Total of HGNC = HGNC**∩**HGNC = 29017

Total of CM = CM**∩**CM = 16821

Input to GoMiner = CM∩HGNC∩BP = 6477
