# Supplementary material for: Functional Categories Associated with Clusters of Genes That Are Co-Expressed across the NCI-60 Cancer Cell Lines
Source: PLoS One. 2012 Jan 24;7(1):e30317. doi: 10.1371/journal.pone.0030317 (PMC3265467; doi:10.1371/journal.pone.0030317)
Supplement: Table S3 — The gross distribution of genes submitted to GoMiner for all 300 clusters. (DOC) [file pone.0030317.s004.doc]

Table S3. The gross distribution of genes submitted to GoMiner for all 300 clusters

| **Cluster number** | **Number of clusters in cuts** | | | |
| --- | --- | --- | --- | --- |
|  | **20** | **40** | **80** | **160** |
| **1** | 237 | 135 | 66 | 66 |
| **2** | 357 | 201 | 59 | 32 |
| **3** | 469 | 147 | 88 | 49 |
| **4** | 256 | 256 | 99 | 38 |
| **5** | 373 | 185 | 91 | 43 |
| **6** | 380 | 178 | 178 | 41 |
| **7** | 326 | 140 | 74 | 41 |
| **8** | 258 | 258 | 109 | 74 |
| **9** | 313 | 190 | 53 | 29 |
| **10** | 346 | 202 | 100 | 42 |
| **11** | 208 | 128 | 89 | 89 |
| **12** | 224 | 101 | 69 | 38 |
| **13** | 443 | 102 | 101 | 31 |
| **14** | 453 | 122 | 57 | 40 |
| **15** | 214 | 86 | 57 | 30 |
| **16** | 347 | 246 | 86 | 23 |
| **17** | 374 | 165 | 74 | 36 |
| **18** | 274 | 215 | 55 | 33 |
| **19** | 454 | 115 | 85 | 39 |
| **20** | 171 | 101 | 85 | 39 |
| **21** | 0 | 204 | 115 | 32 |
| **22** | 0 | 141 | 46 | 31 |
| **23** | 0 | 170 | 94 | 61 |
| **24** | 0 | 238 | 94 | 52 |
| **25** | 0 | 186 | 79 | 42 |
| **26** | 0 | 123 | 59 | 35 |
| **27** | 0 | 217 | 130 | 38 |
| **28** | 0 | 156 | 82 | 31 |
| **29** | 0 | 237 | 73 | 59 |
| **30** | 0 | 132 | 71 | 88 |
| **31** | 0 | 107 | 80 | 48 |
| **32** | 0 | 171 | 186 | 42 |
| **33** | 0 | 132 | 102 | 44 |
| **34** | 0 | 188 | 58 | 47 |
| **35** | 0 | 197 | 116 | 21 |
| **36** | 0 | 157 | 39 | 63 |
| **37** | 0 | 133 | 149 | 58 |
| **38** | 0 | 99 | 101 | 31 |
| **39** | 0 | 102 | 97 | 58 |
| **40** | 0 | 114 | 71 | 33 |
| **41** | 0 | 0 | 103 | 39 |
| **42** | 0 | 0 | 132 | 46 |
| **43** | 0 | 0 | 56 | 39 |
| **44** | 0 | 0 | 79 | 34 |
| **45** | 0 | 0 | 132 | 85 |
| **46** | 0 | 0 | 101 | 53 |
| **47** | 0 | 0 | 65 | 47 |
| **48** | 0 | 0 | 59 | 46 |
| **49** | 0 | 0 | 59 | 24 |
| **50** | 0 | 0 | 80 | 37 |
| **51** | 0 | 0 | 111 | 55 |
| **52** | 0 | 0 | 85 | 83 |
| **53** | 0 | 0 | 75 | 33 |
| **54** | 0 | 0 | 129 | 77 |
| **55** | 0 | 0 | 110 | 38 |
| **56** | 0 | 0 | 83 | 24 |
| **57** | 0 | 0 | 99 | 17 |
| **58** | 0 | 0 | 58 | 29 |
| **59** | 0 | 0 | 57 | 101 |
| **60** | 0 | 0 | 39 | 24 |
| **61** | 0 | 0 | 82 | 29 |
| **62** | 0 | 0 | 88 | 34 |
| **63** | 0 | 0 | 50 | 33 |
| **64** | 0 | 0 | 57 | 33 |
| **65** | 0 | 0 | 86 | 80 |
| **66** | 0 | 0 | 62 | 48 |
| **67** | 0 | 0 | 90 | 48 |
| **68** | 0 | 0 | 51 | 111 |
| **69** | 0 | 0 | 52 | 40 |
| **70** | 0 | 0 | 76 | 41 |
| **71** | 0 | 0 | 85 | 32 |
| **72** | 0 | 0 | 65 | 54 |
| **73** | 0 | 0 | 45 | 35 |
| **74** | 0 | 0 | 50 | 26 |
| **75** | 0 | 0 | 42 | 66 |
| **76** | 0 | 0 | 66 | 33 |
| **77** | 0 | 0 | 52 | 48 |
| **78** | 0 | 0 | 50 | 35 |
| **79** | 0 | 0 | 55 | 31 |
| **80** | 0 | 0 | 44 | 38 |
| **81** | 0 | 0 | 0 | 44 |
| **82** | 0 | 0 | 0 | 58 |
| **83** | 0 | 0 | 0 | 123 |
| **84** | 0 | 0 | 0 | 54 |
| **85** | 0 | 0 | 0 | 18 |
| **86** | 0 | 0 | 0 | 35 |
| **87** | 0 | 0 | 0 | 39 |
| **88** | 0 | 0 | 0 | 42 |
| **89** | 0 | 0 | 0 | 39 |
| **90** | 0 | 0 | 0 | 25 |
| **91** | 0 | 0 | 0 | 34 |
| **92** | 0 | 0 | 0 | 35 |
| **93** | 0 | 0 | 0 | 61 |
| **94** | 0 | 0 | 0 | 24 |
| **95** | 0 | 0 | 0 | 62 |
| **96** | 0 | 0 | 0 | 86 |
| **97** | 0 | 0 | 0 | 32 |
| **98** | 0 | 0 | 0 | 39 |
| **99** | 0 | 0 | 0 | 21 |
| **100** | 0 | 0 | 0 | 31 |
| **101** | 0 | 0 | 0 | 29 |
| **102** | 0 | 0 | 0 | 44 |
| **103** | 0 | 0 | 0 | 48 |
| **104** | 0 | 0 | 0 | 27 |
| **105** | 0 | 0 | 0 | 41 |
| **106** | 0 | 0 | 0 | 18 |
| **107** | 0 | 0 | 0 | 22 |
| **108** | 0 | 0 | 0 | 21 |
| **109** | 0 | 0 | 0 | 43 |
| **110** | 0 | 0 | 0 | 37 |
| **111** | 0 | 0 | 0 | 22 |
| **112** | 0 | 0 | 0 | 40 |
| **113** | 0 | 0 | 0 | 45 |
| **114** | 0 | 0 | 0 | 15 |
| **115** | 0 | 0 | 0 | 33 |
| **116** | 0 | 0 | 0 | 22 |
| **117** | 0 | 0 | 0 | 64 |
| **118** | 0 | 0 | 0 | 23 |
| **119** | 0 | 0 | 0 | 24 |
| **120** | 0 | 0 | 0 | 30 |
| **121** | 0 | 0 | 0 | 50 |
| **122** | 0 | 0 | 0 | 42 |
| **123** | 0 | 0 | 0 | 42 |
| **124** | 0 | 0 | 0 | 22 |
| **125** | 0 | 0 | 0 | 29 |
| **126** | 0 | 0 | 0 | 26 |
| **127** | 0 | 0 | 0 | 26 |
| **128** | 0 | 0 | 0 | 22 |
| **129** | 0 | 0 | 0 | 23 |
| **130** | 0 | 0 | 0 | 25 |
| **131** | 0 | 0 | 0 | 23 |
| **132** | 0 | 0 | 0 | 27 |
| **133** | 0 | 0 | 0 | 22 |
| **134** | 0 | 0 | 0 | 37 |
| **135** | 0 | 0 | 0 | 52 |
| **136** | 0 | 0 | 0 | 38 |
| **137** | 0 | 0 | 0 | 50 |
| **138** | 0 | 0 | 0 | 49 |
| **139** | 0 | 0 | 0 | 26 |
| **140** | 0 | 0 | 0 | 55 |
| **141** | 0 | 0 | 0 | 40 |
| **142** | 0 | 0 | 0 | 40 |
| **143** | 0 | 0 | 0 | 40 |
| **144** | 0 | 0 | 0 | 29 |
| **145** | 0 | 0 | 0 | 25 |
| **146** | 0 | 0 | 0 | 31 |
| **147** | 0 | 0 | 0 | 23 |
| **148** | 0 | 0 | 0 | 30 |
| **149** | 0 | 0 | 0 | 37 |
| **150** | 0 | 0 | 0 | 33 |
| **151** | 0 | 0 | 0 | 53 |
| **152** | 0 | 0 | 0 | 24 |
| **153** | 0 | 0 | 0 | 20 |
| **154** | 0 | 0 | 0 | 35 |
| **155** | 0 | 0 | 0 | 44 |
| **156** | 0 | 0 | 0 | 34 |
| **157** | 0 | 0 | 0 | 22 |
| **158** | 0 | 0 | 0 | 25 |
| **159** | 0 | 0 | 0 | 26 |
| **160** | 0 | 0 | 0 | 37 |
| **Total** | 6477 | 6477 | 6477 | 6477 |
| **Average genes/cluster** | 323 | 161 | 81 | 40 |

The counts in this table are restricted to genes that have HGNC symbols and that map to the GO Biological Process ontology.
