# Supplementary material for: Functional Categories Associated with Clusters of Genes That Are Co-Expressed across the NCI-60 Cancer Cell Lines
Source: PLoS One. 2012 Jan 24;7(1):e30317. doi: 10.1371/journal.pone.0030317 (PMC3265467; doi:10.1371/journal.pone.0030317)
Supplement: Table S4 — Parameters used in HTGM analyses. (DOC) [file pone.0030317.s005.doc]

**Table S4. Parame**ters used in HTGM analyses

| **Parameter** | **Value** |
| --- | --- |
| GO database version | Sept., 2009 |
| GoMiner version | Web HTGM |
| GoMiner build | 248 |
| datasource | UniProtKB |
| organism | human |
| evidence code level | 1 |
| cross reference | false |
| synonym | false |
| FDR threshold | 0.10 |
| p-value threshold | 0.10 |
| randomizations | 100 |
| smallest category size | 5 |
| largest category size | 500 |
| root category | GO:0008150 biological process |
