# Supplementary material for: Functional Categories Associated with Clusters of Genes That Are Co-Expressed across the NCI-60 Cancer Cell Lines
Source: PLoS One. 2012 Jan 24;7(1):e30317. doi: 10.1371/journal.pone.0030317 (PMC3265467; doi:10.1371/journal.pone.0030317)
Supplement: Table S5 — The gross distribution of GO categories resulting from running GoMiner on all 300 clusters. (PDF) [file pone.0030317.s006.pdf]

Table S5. The gross distribution of GO categories resulting from running GoMiner on all 300 clusters

| Cluster number | Number of clusters in cuts | | | |
|---|---|---|---|---|
| | 20 | 40 | 80 | 160 |
| 1 | 0 | 0 | 11 | 12 |
| 2 | 5 | 13 | 18 | 0 |
| 3 | 0 | 0 | 0 | 0 |
| 4 | 27 | 27 | 0 | 0 |
| 5 | 10 | 2 | 0 | 5 |
| 6 | 0 | 0 | 0 | 1 |
| 7 | 3 | 2 | 0 | 0 |
| 8 | 1 | 5 | 30 | 30 |
| 9 | 0 | 0 | 0 | 0 |
| 10 | 40 | 0 | 0 | 0 |
| 11 | 0 | 23 | 12 | 11 |
| 12 | 0 | 0 | 0 | 0 |
| 13 | 7 | 0 | 0 | 1 |
| 14 | 0 | 0 | 0 | 0 |
| 15 | 8 | 0 | 14 | 0 |
| 16 | 24 | 0 | 0 | 0 |
| 17 | 12 | 0 | 17 | 0 |
| 18 | 2 | 0 | 0 | 0 |
| 19 | 0 | 13 | 0 | 0 |
| 20 | 0 | 56 | 0 | 1 |
| 21 | NA | 5 | 12 | 0 |
| 22 | NA | 2 | 17 | 0 |
| 23 | NA | 21 | 3 | 0 |
| 24 | NA | 0 | 0 | 0 |
| 25 | NA | 2 | 0 | 1 |
| 26 | NA | 0 | 92 | 1 |
| 27 | NA | 2 | 1 | 0 |
| 28 | NA | 0 | 26 | 0 |
| 29 | NA | 0 | 0 | 86 |
| 30 | NA | 11 | 1 | 2 |
| 31 | NA | 2 | 0 | 0 |
| 32 | NA | 0 | 7 | 7 |
| 33 | NA | 0 | 0 | 0 |
| 34 | NA | 0 | 0 | 41 |
| 35 | NA | 17 | 0 | 1 |
| 36 | NA | 0 | 0 | 0 |
| 37 | NA | 3 | 0 | 0 |
| 38 | NA | 29 | 15 | 0 |
| 39 | NA | 34 | 0 | 0 |
| 40 | NA | 0 | 0 | 4 |
| 41 | NA | NA | 1 | 0 |
| 42 | NA | NA | 17 | 0 |
| 43 | NA | NA | 12 | 0 |
| 44 | NA | NA | 2 | 0 |
| 45 | NA | NA | 0 | 13 |
| 46 | NA | NA | 3 | 0 |
| 47 | NA | NA | 0 | 0 |
| 48 | NA | NA | 0 | 0 |
| 49 | NA | NA | 2 | 14 |
| 50 | NA | NA | 0 | 0 |
| 51 | NA | NA | 29 | 1 |
| 52 | NA | NA | 0 | 60 |
| 53 | NA | NA | 2 | 20 |
| 54 | NA | NA | 0 | 0 |
| 55 | NA | NA | 7 | 0 |
| 56 | NA | NA | 0 | 0 |
| 57 | NA | NA | 26 | 0 |
| 58 | NA | NA | 0 | 2 |
| 59 | NA | NA | 0 | 2 |
| 60 | NA | NA | 0 | 0 |
| 61 | NA | NA | 0 | 0 |
| 62 | NA | NA | 0 | 2 |
| 63 | NA | NA | 0 | 0 |
| 64 | NA | NA | 0 | 0 |
| 65 | NA | NA | 0 | 0 |
| 66 | NA | NA | 0 | 2 |
| 67 | NA | NA | 0 | 0 |
| 68 | NA | NA | 0 | 32 |
| 69 | NA | NA | 2 | 11 |
| 70 | NA | NA | 0 | 4 |
| 71 | NA | NA | 0 | 1 |
| 72 | NA | NA | 1 | 22 |
| 73 | NA | NA | 0 | 0 |
| 74 | NA | NA | 4 | 0 |
| 75 | NA | NA | 5 | 0 |
| 76 | NA | NA | 2 | 0 |
| 77 | NA | NA | 2 | 1 |
| 78 | NA | NA | 22 | 0 |
| 79 | NA | NA | 36 | 0 |
| 80 | NA | NA | 19 | 18 |
| 81 | NA | NA | NA | 11 |
| 82 | NA | NA | NA | 0 |
| 83 | NA | NA | NA | 1 |
| 84 | NA | NA | NA | 12 |
| 85 | NA | NA | NA | 0 |
| 86 | NA | NA | NA | 0 |
| 87 | NA | NA | NA | 0 |
| 88 | NA | NA | NA | 0 |
| 89 | NA | NA | NA | 0 |
| 90 | NA | NA | NA | 0 |
| 91 | NA | NA | NA | 0 |
| 92 | NA | NA | NA | 0 |
| 93 | NA | NA | NA | 0 |
| 94 | NA | NA | NA | 0 |
| 95 | NA | NA | NA | 36 |
| 96 | NA | NA | NA | 0 |
| 97 | NA | NA | NA | 6 |
| 98 | NA | NA | NA | 1 |
| 99 | NA | NA | NA | 0 |
| 100 | NA | NA | NA | 0 |
| 101 | NA | NA | NA | 0 |
| 102 | NA | NA | NA | 0 |
| 103 | NA | NA | NA | 0 |
| 104 | NA | NA | NA | 86 |
| 105 | NA | NA | NA | 9 |
| 106 | NA | NA | NA | 0 |
| 107 | NA | NA | NA | 0 |
| 108 | NA | NA | NA | 0 |
| 109 | NA | NA | NA | 30 |
| 110 | NA | NA | NA | 0 |
| 111 | NA | NA | NA | 0 |
| 112 | NA | NA | NA | 1 |
| 113 | NA | NA | NA | 0 |
| 114 | NA | NA | NA | 21 |
| 115 | NA | NA | NA | 0 |
| 116 | NA | NA | NA | 0 |
| 117 | NA | NA | NA | 1 |
| 118 | NA | NA | NA | 0 |
| 119 | NA | NA | NA | 0 |
| 120 | NA | NA | NA | 0 |
| 121 | NA | NA | NA | 2 |
| 122 | NA | NA | NA | 4 |
| 123 | NA | NA | NA | 5 |
| 124 | NA | NA | NA | 1 |
| 125 | NA | NA | NA | 0 |
| 126 | NA | NA | NA | 0 |
| 127 | NA | NA | NA | 0 |
| 128 | NA | NA | NA | 0 |
| 129 | NA | NA | NA | 0 |
| 130 | NA | NA | NA | 3 |
| 131 | NA | NA | NA | 0 |
| 132 | NA | NA | NA | 20 |
| 133 | NA | NA | NA | 0 |
| 134 | NA | NA | NA | 23 |
| 135 | NA | NA | NA | 3 |
| 136 | NA | NA | NA | 0 |
| 137 | NA | NA | NA | 21 |
| 138 | NA | NA | NA | 0 |
| 139 | NA | NA | NA | 1 |
| 140 | NA | NA | NA | 5 |
| 141 | NA | NA | NA | 0 |
| 142 | NA | NA | NA | 7 |
| 143 | NA | NA | NA | 9 |
| 144 | NA | NA | NA | 1 |
| 145 | NA | NA | NA | 0 |
| 146 | NA | NA | NA | 0 |
| 147 | NA | NA | NA | 0 |
| 148 | NA | NA | NA | 1 |
| 149 | NA | NA | NA | 1 |
| 150 | NA | NA | NA | 0 |
| 151 | NA | NA | NA | 2 |
| 152 | NA | NA | NA | 0 |
| 153 | NA | NA | NA | 0 |
| 154 | NA | NA | NA | 23 |
| 155 | NA | NA | NA | 20 |
| 156 | NA | NA | NA | 0 |
| 157 | NA | NA | NA | 7 |
| 158 | NA | NA | NA | 0 |
| 159 | NA | NA | NA | 2 |
| 160 | NA | NA | NA | 9 |
| Total | 139 | 269 | 470 | 794 |
| Fraction of clusters having at least one category | 0.55 | 0.47 | 0.42 | 0.41 |

"NA" indicates that the cluster number was not part of the cut. For example, in the column for the 20 cut, row 21 is designated as "NA" since there was no cluster 21 in that cut.

The count of categories given here is based upon the HTGM .report file, which, in contrast to the CIMs, is not filtered for large generic categories. In principle, as the number of clusters in a cut increases, there are three competing factors at play in determining the number of HTGM categories:

1. the statistical power of HTGM is decreased as the number of genes *per* cluster decreases
2. noise, and therefore spurious categories, might increase as the number of genes *per* cluster decreases
3. the genes in a cluster are "purified" in that "contaminating" genes might be removed

We find that in fact the total number of HTGM categories increases roughly proportionately so apparently (2) and (3) predominate. But that trend does not continue indefinitely, since the total for a 320 cut set of clusters (data not shown) is 1,086.
