# Supplementary material for: Functional Categories Associated with Clusters of Genes That Are Co-Expressed across the NCI-60 Cancer Cell Lines
Source: PLoS One. 2012 Jan 24;7(1):e30317. doi: 10.1371/journal.pone.0030317 (PMC3265467; doi:10.1371/journal.pone.0030317)
Supplement: Table S6 — Distribution of scores. (DOC) [file pone.0030317.s007.doc]

Table S6. Distribution of scores

| **Score** | **Number of categories** | **Fraction of categories** |
| --- | --- | --- |
| **1111** | 59 | 0.096 |
| **1110** | 46 | 0.075 |
| **1101** | 2 | 0.003 |
| **1100** | 156 | 0.256 |
| **1010** | 5 | 0.008 |
| **1001** | 6 | 0.009 |
| **1000** | 243 | 0.399 |
| **0111** | 3 | 0.004 |
| **0110** | 24 | 0.039 |
| **0101** | 1 | 0.001 |
| **0100** | 27 | 0.044 |
| **0011** | 6 | 0.009 |
| **0010** | 20 | 0.032 |
| **0001** | 11 | 0.018 |
| **Total** | 609 | 1.000 |

The scores are sorted from best to worst. The best case “1111” represents a category that appears in each of the four cuts within a single cluster group. A next-to-worst case “0001” represents a category that appears in only the 20 cut (least significant bit corresponds to lowest-numbered cut); the category appears in no other cuts in any cluster group. The worst case score “0000” represents no significant GO categories in any cut, and constitutes the majority of the cases; those instances are not explicitly considered here.
