# Supplementary material for: Functional Categories Associated with Clusters of Genes That Are Co-Expressed across the NCI-60 Cancer Cell Lines
Source: PLoS One. 2012 Jan 24;7(1):e30317. doi: 10.1371/journal.pone.0030317 (PMC3265467; doi:10.1371/journal.pone.0030317)
Supplement: Table S7 — Categories according to score. (DOC) [file pone.0030317.s008.doc]

Table S7. Categories according to score

| **0100** | **0101** | **0110** | **0111** | **1100** | **1101** | **1110** | **1111** | **0001** | **0010** | **0011** | **1000** | **1001** | **1010** |
| --- | --- | --- | --- | --- | --- | --- | --- | --- | --- | --- | --- | --- | --- |
| GO:0060389_pathway-restricted_SMAD_protein_phosphorylation | GO:0006570_tyrosine_metabolic_process | GO:0051785_positive_regulation_of_nuclear_division | GO:0051353_positive_regulation_of_oxidoreductase_activity | GO:0070668_positive_regulation_of_mast_cell_proliferation | GO:0034622_cellular_macromolecular_complex_assembly | GO:0051249_regulation_of_lymphocyte_activation | GO:0070647_protein_modification_by_small_protein_conjugation_or_removal | GO:0055072_iron_ion_homeostasis | GO:0060048_cardiac_muscle_contraction | GO:0050954_sensory_perception_of_mechanical_stimulus | GO:0070838_divalent_metal_ion_transport | GO:0051271_negative_regulation_of_cell_motion | GO:0044085_cellular_component_biogenesis |
| GO:0051298_centrosome_duplication |  | GO:0051341_regulation_of_oxidoreductase_activity | GO:0048066_pigmentation_during_development | GO:0070227_lymphocyte_apoptosis | GO:0034621_cellular_macromolecular_complex_subunit_organization | GO:0050865_regulation_of_cell_activation | GO:0051704_multi-organism_process | GO:0048864_stem_cell_development | GO:0051318_G1_phase | GO:0050878_regulation_of_body_fluid_levels | GO:0070666_regulation_of_mast_cell_proliferation | GO:0045596_negative_regulation_of_cell_differentiation | GO:0043933_macromolecular_complex_subunit_organization |
| GO:0051239_regulation_of_multicellular_organismal_process |  | GO:0051046_regulation_of_secretion | GO:0034984_cellular_response_to_DNA_damage_stimulus | GO:0070198_protein_localization_to_telomere |  | GO:0048869_cellular_developmental_process | GO:0051674_localization_of_cell | GO:0048863_stem_cell_differentiation | GO:0051172_negative_regulation_of_nitrogen_compound_metabolic_process | GO:0042060_wound_healing | GO:0070665_positive_regulation_of_leukocyte_proliferation | GO:0032879_regulation_of_localization | GO:0042147_retrograde_transport__endosome_to_Golgi |
| GO:0051235_maintenance_of_location |  | GO:0045840_positive_regulation_of_mitosis |  | GO:0065004_protein-DNA_complex_assembly |  | GO:0048285_organelle_fission | GO:0051270_regulation_of_cell_motion | GO:0030168_platelet_activation | GO:0050817_coagulation | GO:0007605_sensory_perception_of_sound | GO:0070663_regulation_of_leukocyte_proliferation | GO:0030336_negative_regulation_of_cell_migration | GO:0034728_nucleosome_organization |
| GO:0050962_detection_of_light_stimulus_involved_in_sensory_perception |  | GO:0042440_pigment_metabolic_process |  | GO:0065003_macromolecular_complex_assembly |  | GO:0045076_regulation_of_interleukin-2_biosynthetic_process | GO:0051056_regulation_of_small_GTPase_mediated_signal_transduction | GO:0019827_stem_cell_maintenance | GO:0043039_tRNA_aminoacylation | GO:0007599_hemostasis | GO:0070662_mast_cell_proliferation | GO:0022604_regulation_of_cell_morphogenesis | GO:0031497_chromatin_assembly |
| GO:0050908_detection_of_light_stimulus_involved_in_visual_perception |  | GO:0042108_positive_regulation_of_cytokine_biosynthetic_process |  | GO:0060558_regulation_of_calcidiol_1-monooxygenase_activity |  | GO:0043087_regulation_of_GTPase_activity | GO:0050808_synapse_organization | GO:0008360_regulation_of_cell_shape | GO:0043038_amino_acid_activation | GO:0006281_DNA_repair | GO:0070661_leukocyte_proliferation | GO:0007059_chromosome_segregation |  |
| GO:0050906_detection_of_stimulus_involved_in_sensory_perception |  | GO:0032770_positive_regulation_of_monooxygenase_activity |  | GO:0060548_negative_regulation_of_cell_death |  | GO:0043062_extracellular_structure_organization | GO:0048870_cell_motility | GO:0007010_cytoskeleton_organization | GO:0042770_DNA_damage_response__signal_transduction |  | GO:0070555_response_to_interleukin-1 |  |  |
| GO:0046148_pigment_biosynthetic_process |  | GO:0032663_regulation_of_interleukin-2_production |  | GO:0060249_anatomical_structure_homeostasis |  | GO:0042094_interleukin-2_biosynthetic_process | GO:0048069_eye_pigmentation | GO:0006826_iron_ion_transport | GO:0033554_cellular_response_to_stress |  | GO:0060402_calcium_ion_transport_into_cytosol |  |  |
| GO:0045333_cellular_respiration |  | GO:0032623_interleukin-2_production |  | GO:0055082_cellular_chemical_homeostasis |  | GO:0034470_ncRNA_processing | GO:0046649_lymphocyte_activation | GO:0006091_generation_of_precursor_metabolites_and_energy | GO:0032768_regulation_of_monooxygenase_activity |  | GO:0060401_cytosolic_calcium_ion_transport |  |  |
| GO:0043550_regulation_of_lipid_kinase_activity |  | GO:0032297_negative_regulation_of_DNA_replication_initiation |  | GO:0051276_chromosome_organization |  | GO:0034330_cell_junction_organization | GO:0046578_regulation_of_Ras_protein_signal_transduction | GO:0006024_glycosaminoglycan_biosynthetic_process | GO:0019083_viral_transcription |  | GO:0060193_positive_regulation_of_lipase_activity |  |  |
| GO:0030317_sperm_motility |  | GO:0031570_DNA_integrity_checkpoint |  | GO:0051253_negative_regulation_of_RNA_metabolic_process |  | GO:0032319_regulation_of_Rho_GTPase_activity | GO:0045595_regulation_of_cell_differentiation | GO:0000165_MAPKKK_cascade | GO:0019080_viral_genome_expression |  | GO:0060191_regulation_of_lipase_activity |  |  |
| GO:0019079_viral_genome_replication |  | GO:0031327_negative_regulation_of_cellular_biosynthetic_process |  | GO:0051250_negative_regulation_of_lymphocyte_activation |  | GO:0032318_regulation_of_Ras_GTPase_activity | GO:0045321_leukocyte_activation |  | GO:0009967_positive_regulation_of_signal_transduction |  | GO:0055114_oxidation_reduction |  |  |
| GO:0015980_energy_derivation_by_oxidation_of_organic_compounds |  | GO:0031324_negative_regulation_of_cellular_metabolic_process |  | GO:0051248_negative_regulation_of_protein_metabolic_process |  | GO:0032205_negative_regulation_of_telomere_maintenance | GO:0045086_positive_regulation_of_interleukin-2_biosynthetic_process |  | GO:0008016_regulation_of_heart_contraction |  | GO:0055085_transmembrane_transport |  |  |
| GO:0010862_positive_regulation_of_pathway-restricted_SMAD_protein_phosphorylation |  | GO:0030174_regulation_of_DNA_replication_initiation |  | GO:0051246_regulation_of_protein_metabolic_process |  | GO:0032200_telomere_organization | GO:0045058_T_cell_selection |  | GO:0007596_blood_coagulation |  | GO:0055080_cation_homeostasis |  |  |
| GO:0010564_regulation_of_cell_cycle_process |  | GO:0016052_carbohydrate_catabolic_process |  | GO:0051093_negative_regulation_of_developmental_process |  | GO:0030217_T_cell_differentiation | GO:0043631_RNA_polyadenylation |  | GO:0007064_mitotic_sister_chromatid_cohesion |  | GO:0055074_calcium_ion_homeostasis |  |  |
| GO:0010557_positive_regulation_of_macromolecule_biosynthetic_process |  | GO:0010605_negative_regulation_of_macromolecule_metabolic_process |  | GO:0051053_negative_regulation_of_DNA_metabolic_process |  | GO:0030147_natriuresis | GO:0043588_skin_development |  | GO:0006974_response_to_DNA_damage_stimulus |  | GO:0055066_di-__tri-valent_inorganic_cation_homeostasis |  |  |
| GO:0009892_negative_regulation_of_metabolic_process |  | GO:0010558_negative_regulation_of_macromolecule_biosynthetic_process |  | GO:0051048_negative_regulation_of_secretion |  | GO:0030098_lymphocyte_differentiation | GO:0042110_T_cell_activation |  | GO:0006418_tRNA_aminoacylation_for_protein_translation |  | GO:0055065_metal_ion_homeostasis |  |  |
| GO:0009584_detection_of_visible_light |  | GO:0009890_negative_regulation_of_biosynthetic_process |  | GO:0050909_sensory_perception_of_taste |  | GO:0022610_biological_adhesion | GO:0040012_regulation_of_locomotion |  | GO:0006367_transcription_initiation_from_RNA_polymerase_II_promoter |  | GO:0051924_regulation_of_calcium_ion_transport |  |  |
| GO:0009583_detection_of_light_stimulus |  | GO:0009611_response_to_wounding |  | GO:0050890_cognition |  | GO:0022414_reproductive_process | GO:0035023_regulation_of_Rho_protein_signal_transduction |  | GO:0006301_postreplication_repair |  | GO:0051789_response_to_protein_stimulus |  |  |
| GO:0009582_detection_of_abiotic_stimulus |  | GO:0006954_inflammatory_response |  | GO:0050877_neurological_system_process |  | GO:0019226_transmission_of_nerve_impulse | GO:0033627_cell_adhesion_mediated_by_integrin |  | GO:0000080_G1_phase_of_mitotic_cell_cycle |  | GO:0051707_response_to_other_organism |  |  |
| GO:0009581_detection_of_external_stimulus |  | GO:0006383_transcription_from_RNA_polymerase_III_promoter |  | GO:0050866_negative_regulation_of_cell_activation |  | GO:0016072_rRNA_metabolic_process | GO:0032446_protein_modification_by_small_protein_conjugation |  |  |  | GO:0051592_response_to_calcium_ion |  |  |
| GO:0007602_phototransduction |  | GO:0006352_transcription_initiation |  | GO:0050864_regulation_of_B_cell_activation |  | GO:0009888_tissue_development | GO:0031124_mRNA_3'-end_processing |  |  |  | GO:0051480_cytosolic_calcium_ion_homeostasis |  |  |
| GO:0007098_centrosome_cycle |  | GO:0002673_regulation_of_acute_inflammatory_response |  | GO:0050851_antigen_receptor-mediated_signaling_pathway |  | GO:0009410_response_to_xenobiotic_stimulus | GO:0030334_regulation_of_cell_migration |  |  |  | GO:0051345_positive_regulation_of_hydrolase_activity |  |  |
| GO:0007052_mitotic_spindle_organization |  | GO:0000076_DNA_replication_checkpoint |  | GO:0050801_ion_homeostasis |  | GO:0008299_isoprenoid_biosynthetic_process | GO:0030199_collagen_fibril_organization |  |  |  | GO:0051327_M_phase_of_meiotic_cell_cycle |  |  |
| GO:0007020_microtubule_nucleation |  |  |  | GO:0050792_regulation_of_viral_reproduction |  | GO:0007507_heart_development | GO:0030198_extracellular_matrix_organization |  |  |  | GO:0051321_meiotic_cell_cycle |  |  |
| GO:0005975_carbohydrate_metabolic_process |  |  |  | GO:0050778_positive_regulation_of_immune_response |  | GO:0007398_ectoderm_development | GO:0030036_actin_cytoskeleton_organization |  |  |  | GO:0051251_positive_regulation_of_lymphocyte_activation |  |  |
| GO:0000272_polysaccharide_catabolic_process |  |  |  | GO:0050776_regulation_of_immune_response |  | GO:0007268_synaptic_transmission | GO:0030029_actin_filament-based_process |  |  |  | GO:0051240_positive_regulation_of_multicellular_organismal_process |  |  |
|  |  |  |  | GO:0050730_regulation_of_peptidyl-tyrosine_phosphorylation |  | GO:0007267_cell-cell_signaling | GO:0022618_ribonucleoprotein_complex_assembly |  |  |  | GO:0051236_establishment_of_RNA_localization |  |  |
|  |  |  |  | GO:0050691_regulation_of_defense_response_to_virus_by_host |  | GO:0007266_Rho_protein_signal_transduction | GO:0022613_ribonucleoprotein_complex_biogenesis |  |  |  | GO:0051169_nuclear_transport |  |  |
|  |  |  |  | GO:0050688_regulation_of_defense_response_to_virus |  | GO:0007155_cell_adhesion | GO:0016567_protein_ubiquitination |  |  |  | GO:0051128_regulation_of_cellular_component_organization |  |  |
|  |  |  |  | GO:0050671_positive_regulation_of_lymphocyte_proliferation |  | GO:0007067_mitosis | GO:0016477_cell_migration |  |  |  | GO:0051099_positive_regulation_of_binding |  |  |
|  |  |  |  | GO:0048534_hemopoietic_or_lymphoid_organ_development |  | GO:0007062_sister_chromatid_cohesion | GO:0016339_calcium-dependent_cell-cell_adhesion |  |  |  | GO:0051094_positive_regulation_of_developmental_process |  |  |
|  |  |  |  | GO:0048525_negative_regulation_of_viral_reproduction |  | GO:0007049_cell_cycle | GO:0016337_cell-cell_adhesion |  |  |  | GO:0051052_regulation_of_DNA_metabolic_process |  |  |
|  |  |  |  | GO:0048511_rhythmic_process |  | GO:0006952_defense_response | GO:0016071_mRNA_metabolic_process |  |  |  | GO:0051050_positive_regulation_of_transport |  |  |
|  |  |  |  | GO:0045947_negative_regulation_of_translational_initiation |  | GO:0006805_xenobiotic_metabolic_process | GO:0010646_regulation_of_cell_communication |  |  |  | GO:0051049_regulation_of_transport |  |  |
|  |  |  |  | GO:0045937_positive_regulation_of_phosphate_metabolic_process |  | GO:0006364_rRNA_processing | GO:0009966_regulation_of_signal_transduction |  |  |  | GO:0051047_positive_regulation_of_secretion |  |  |
|  |  |  |  | GO:0045597_positive_regulation_of_cell_differentiation |  | GO:0006261_DNA-dependent_DNA_replication | GO:0008380_RNA_splicing |  |  |  | GO:0051004_regulation_of_lipoprotein_lipase_activity |  |  |
|  |  |  |  | GO:0045090_retroviral_genome_replication |  | GO:0002694_regulation_of_leukocyte_activation | GO:0007565_female_pregnancy |  |  |  | GO:0050994_regulation_of_lipid_catabolic_process |  |  |
|  |  |  |  | GO:0045088_regulation_of_innate_immune_response |  | GO:0002684_positive_regulation_of_immune_system_process | GO:0007416_synaptogenesis |  |  |  | GO:0050880_regulation_of_blood_vessel_size |  |  |
|  |  |  |  | GO:0045071_negative_regulation_of_viral_genome_replication |  | GO:0002520_immune_system_development | GO:0007399_nervous_system_development |  |  |  | GO:0050870_positive_regulation_of_T_cell_activation |  |  |
|  |  |  |  | GO:0045069_regulation_of_viral_genome_replication |  | GO:0001501_skeletal_system_development | GO:0007265_Ras_protein_signal_transduction |  |  |  | GO:0050867_positive_regulation_of_cell_activation |  |  |
|  |  |  |  | GO:0045017_glycerolipid_biosynthetic_process |  | GO:0000723_telomere_maintenance | GO:0006959_humoral_immune_response |  |  |  | GO:0050863_regulation_of_T_cell_activation |  |  |
|  |  |  |  | GO:0043069_negative_regulation_of_programmed_cell_death |  | GO:0000280_nuclear_division | GO:0006955_immune_response |  |  |  | GO:0050714_positive_regulation_of_protein_secretion |  |  |
|  |  |  |  | GO:0043068_positive_regulation_of_programmed_cell_death |  | GO:0000278_mitotic_cell_cycle | GO:0006928_cell_motion |  |  |  | GO:0050708_regulation_of_protein_secretion |  |  |
|  |  |  |  | GO:0043067_regulation_of_programmed_cell_death |  | GO:0000087_M_phase_of_mitotic_cell_cycle | GO:0006414_translational_elongation |  |  |  | GO:0050670_regulation_of_lymphocyte_proliferation |  |  |
|  |  |  |  | GO:0043066_negative_regulation_of_apoptosis |  | GO:0000003_reproduction | GO:0006397_mRNA_processing |  |  |  | GO:0050658_RNA_transport |  |  |
|  |  |  |  | GO:0043065_positive_regulation_of_apoptosis |  |  | GO:0006396_RNA_processing |  |  |  | GO:0050657_nucleic_acid_transport |  |  |
|  |  |  |  | GO:0042981_regulation_of_apoptosis |  |  | GO:0006378_mRNA_polyadenylation |  |  |  | GO:0050650_chondroitin_sulfate_proteoglycan_biosynthetic_process |  |  |
|  |  |  |  | GO:0042516_regulation_of_tyrosine_phosphorylation_of_Stat3_protein |  |  | GO:0006376_mRNA_splice_site_selection |  |  |  | GO:0048878_chemical_homeostasis |  |  |
|  |  |  |  | GO:0042509_regulation_of_tyrosine_phosphorylation_of_STAT_protein |  |  | GO:0006302_double-strand_break_repair |  |  |  | GO:0048771_tissue_remodeling |  |  |
|  |  |  |  | GO:0042503_tyrosine_phosphorylation_of_Stat3_protein |  |  | GO:0006260_DNA_replication |  |  |  | GO:0048730_epidermis_morphogenesis |  |  |
|  |  |  |  | GO:0042445_hormone_metabolic_process |  |  | GO:0006259_DNA_metabolic_process |  |  |  | GO:0048706_embryonic_skeletal_system_development |  |  |
|  |  |  |  | GO:0042327_positive_regulation_of_phosphorylation |  |  | GO:0002682_regulation_of_immune_system_process |  |  |  | GO:0048699_generation_of_neurons |  |  |
|  |  |  |  | GO:0042269_regulation_of_natural_killer_cell_mediated_cytotoxicity |  |  | GO:0002376_immune_system_process |  |  |  | GO:0048666_neuron_development |  |  |
|  |  |  |  | GO:0042267_natural_killer_cell_mediated_cytotoxicity |  |  | GO:0001775_cell_activation |  |  |  | GO:0048646_anatomical_structure_formation_involved_in_morphogenesis |  |  |
|  |  |  |  | GO:0042254_ribosome_biogenesis |  |  | GO:0000398_nuclear_mRNA_splicing__via_spliceosome |  |  |  | GO:0048584_positive_regulation_of_response_to_stimulus |  |  |
|  |  |  |  | GO:0042129_regulation_of_T_cell_proliferation |  |  | GO:0000377_RNA_splicing__via_transesterification_reactions_with_bulged_adenosine_as_nucleophile |  |  |  | GO:0048468_cell_development |  |  |
|  |  |  |  | GO:0042113_B_cell_activation |  |  | GO:0000375_RNA_splicing__via_transesterification_reactions |  |  |  | GO:0048259_regulation_of_receptor-mediated_endocytosis |  |  |
|  |  |  |  | GO:0042107_cytokine_metabolic_process |  |  | GO:0000245_spliceosome_assembly |  |  |  | GO:0048015_phosphoinositide-mediated_signaling |  |  |
|  |  |  |  | GO:0042100_B_cell_proliferation |  |  |  |  |  |  | GO:0046907_intracellular_transport |  |  |
|  |  |  |  | GO:0042089_cytokine_biosynthetic_process |  |  |  |  |  |  | GO:0046651_lymphocyte_proliferation |  |  |
|  |  |  |  | GO:0042035_regulation_of_cytokine_biosynthetic_process |  |  |  |  |  |  | GO:0046634_regulation_of_alpha-beta_T_cell_activation |  |  |
|  |  |  |  | GO:0034754_cellular_hormone_metabolic_process |  |  |  |  |  |  | GO:0046632_alpha-beta_T_cell_differentiation |  |  |
|  |  |  |  | GO:0034660_ncRNA_metabolic_process |  |  |  |  |  |  | GO:0046631_alpha-beta_T_cell_activation |  |  |
|  |  |  |  | GO:0034502_protein_localization_to_chromosome |  |  |  |  |  |  | GO:0046503_glycerolipid_catabolic_process |  |  |
|  |  |  |  | GO:0033036_macromolecule_localization |  |  |  |  |  |  | GO:0046496_nicotinamide_nucleotide_metabolic_process |  |  |
|  |  |  |  | GO:0032845_negative_regulation_of_homeostatic_process |  |  |  |  |  |  | GO:0046394_carboxylic_acid_biosynthetic_process |  |  |
|  |  |  |  | GO:0032675_regulation_of_interleukin-6_production |  |  |  |  |  |  | GO:0046058_cAMP_metabolic_process |  |  |
|  |  |  |  | GO:0032635_interleukin-6_production |  |  |  |  |  |  | GO:0045934_negative_regulation_of_nucleobase__nucleoside__nucleotide_and_nucleic_acid_metabolic_process |  |  |
|  |  |  |  | GO:0032271_regulation_of_protein_polymerization |  |  |  |  |  |  | GO:0045892_negative_regulation_of_transcription__DNA-dependent |  |  |
|  |  |  |  | GO:0032269_negative_regulation_of_cellular_protein_metabolic_process |  |  |  |  |  |  | GO:0045833_negative_regulation_of_lipid_metabolic_process |  |  |
|  |  |  |  | GO:0032268_regulation_of_cellular_protein_metabolic_process |  |  |  |  |  |  | GO:0045806_negative_regulation_of_endocytosis |  |  |
|  |  |  |  | GO:0032204_regulation_of_telomere_maintenance |  |  |  |  |  |  | GO:0045651_positive_regulation_of_macrophage_differentiation |  |  |
|  |  |  |  | GO:0031341_regulation_of_cell_killing |  |  |  |  |  |  | GO:0045649_regulation_of_macrophage_differentiation |  |  |
|  |  |  |  | GO:0031123_RNA_3'-end_processing |  |  |  |  |  |  | GO:0045639_positive_regulation_of_myeloid_cell_differentiation |  |  |
|  |  |  |  | GO:0030888_regulation_of_B_cell_proliferation |  |  |  |  |  |  | GO:0045638_negative_regulation_of_myeloid_cell_differentiation |  |  |
|  |  |  |  | GO:0030154_cell_differentiation |  |  |  |  |  |  | GO:0045637_regulation_of_myeloid_cell_differentiation |  |  |
|  |  |  |  | GO:0030099_myeloid_cell_differentiation |  |  |  |  |  |  | GO:0045184_establishment_of_protein_localization |  |  |
|  |  |  |  | GO:0030097_hemopoiesis |  |  |  |  |  |  | GO:0044255_cellular_lipid_metabolic_process |  |  |
|  |  |  |  | GO:0022403_cell_cycle_phase |  |  |  |  |  |  | GO:0044093_positive_regulation_of_molecular_function |  |  |
|  |  |  |  | GO:0022402_cell_cycle_process |  |  |  |  |  |  | GO:0043900_regulation_of_multi-organism_process |  |  |
|  |  |  |  | GO:0019935_cyclic-nucleotide-mediated_signaling |  |  |  |  |  |  | GO:0043603_cellular_amide_metabolic_process |  |  |
|  |  |  |  | GO:0019724_B_cell_mediated_immunity |  |  |  |  |  |  | GO:0043583_ear_development |  |  |
|  |  |  |  | GO:0019439_aromatic_compound_catabolic_process |  |  |  |  |  |  | GO:0043542_endothelial_cell_migration |  |  |
|  |  |  |  | GO:0019221_cytokine-mediated_signaling_pathway |  |  |  |  |  |  | GO:0043473_pigmentation |  |  |
|  |  |  |  | GO:0018212_peptidyl-tyrosine_modification |  |  |  |  |  |  | GO:0043436_oxoacid_metabolic_process |  |  |
|  |  |  |  | GO:0018108_peptidyl-tyrosine_phosphorylation |  |  |  |  |  |  | GO:0043269_regulation_of_ion_transport |  |  |
|  |  |  |  | GO:0016064_immunoglobulin_mediated_immune_response |  |  |  |  |  |  | GO:0043085_positive_regulation_of_catalytic_activity |  |  |
|  |  |  |  | GO:0010942_positive_regulation_of_cell_death |  |  |  |  |  |  | GO:0043009_chordate_embryonic_development |  |  |
|  |  |  |  | GO:0010941_regulation_of_cell_death |  |  |  |  |  |  | GO:0042692_muscle_cell_differentiation |  |  |
|  |  |  |  | GO:0010833_telomere_maintenance_via_telomere_lengthening |  |  |  |  |  |  | GO:0042304_regulation_of_fatty_acid_biosynthetic_process |  |  |
|  |  |  |  | GO:0010817_regulation_of_hormone_levels |  |  |  |  |  |  | GO:0042102_positive_regulation_of_T_cell_proliferation |  |  |
|  |  |  |  | GO:0010647_positive_regulation_of_cell_communication |  |  |  |  |  |  | GO:0042098_T_cell_proliferation |  |  |
|  |  |  |  | GO:0010604_positive_regulation_of_macromolecule_metabolic_process |  |  |  |  |  |  | GO:0042036_negative_regulation_of_cytokine_biosynthetic_process |  |  |
|  |  |  |  | GO:0010565_regulation_of_cellular_ketone_metabolic_process |  |  |  |  |  |  | GO:0040011_locomotion |  |  |
|  |  |  |  | GO:0010562_positive_regulation_of_phosphorus_metabolic_process |  |  |  |  |  |  | GO:0035239_tube_morphogenesis |  |  |
|  |  |  |  | GO:0010038_response_to_metal_ion |  |  |  |  |  |  | GO:0035150_regulation_of_tube_size |  |  |
|  |  |  |  | GO:0010035_response_to_inorganic_substance |  |  |  |  |  |  | GO:0034447_very-low-density_lipoprotein_particle_clearance |  |  |
|  |  |  |  | GO:0009893_positive_regulation_of_metabolic_process |  |  |  |  |  |  | GO:0034440_lipid_oxidation |  |  |
|  |  |  |  | GO:0009636_response_to_toxin |  |  |  |  |  |  | GO:0034381_lipoprotein_particle_clearance |  |  |
|  |  |  |  | GO:0009605_response_to_external_stimulus |  |  |  |  |  |  | GO:0034329_cell_junction_assembly |  |  |
|  |  |  |  | GO:0009451_RNA_modification |  |  |  |  |  |  | GO:0033700_phospholipid_efflux |  |  |
|  |  |  |  | GO:0009056_catabolic_process |  |  |  |  |  |  | GO:0033559_unsaturated_fatty_acid_metabolic_process |  |  |
|  |  |  |  | GO:0008202_steroid_metabolic_process |  |  |  |  |  |  | GO:0033280_response_to_vitamin_D |  |  |
|  |  |  |  | GO:0008104_protein_localization |  |  |  |  |  |  | GO:0033044_regulation_of_chromosome_organization |  |  |
|  |  |  |  | GO:0007608_sensory_perception_of_smell |  |  |  |  |  |  | GO:0033002_muscle_cell_proliferation |  |  |
|  |  |  |  | GO:0007606_sensory_perception_of_chemical_stimulus |  |  |  |  |  |  | GO:0032984_macromolecular_complex_disassembly |  |  |
|  |  |  |  | GO:0007600_sensory_perception |  |  |  |  |  |  | GO:0032964_collagen_biosynthetic_process |  |  |
|  |  |  |  | GO:0007586_digestion |  |  |  |  |  |  | GO:0032946_positive_regulation_of_mononuclear_cell_proliferation |  |  |
|  |  |  |  | GO:0007517_muscle_organ_development |  |  |  |  |  |  | GO:0032944_regulation_of_mononuclear_cell_proliferation |  |  |
|  |  |  |  | GO:0007417_central_nervous_system_development |  |  |  |  |  |  | GO:0032943_mononuclear_cell_proliferation |  |  |
|  |  |  |  | GO:0007259_JAK-STAT_cascade |  |  |  |  |  |  | GO:0032844_regulation_of_homeostatic_process |  |  |
|  |  |  |  | GO:0007200_activation_of_phospholipase_C_activity_by_G-protein_coupled_receptor_protein_signaling_pathway_coupled_to_IP3_second_messenger |  |  |  |  |  |  | GO:0032787_monocarboxylic_acid_metabolic_process |  |  |
|  |  |  |  | GO:0007186_G-protein_coupled_receptor_protein_signaling_pathway |  |  |  |  |  |  | GO:0032374_regulation_of_cholesterol_transport |  |  |
|  |  |  |  | GO:0007093_mitotic_cell_cycle_checkpoint |  |  |  |  |  |  | GO:0032371_regulation_of_sterol_transport |  |  |
|  |  |  |  | GO:0007051_spindle_organization |  |  |  |  |  |  | GO:0032369_negative_regulation_of_lipid_transport |  |  |
|  |  |  |  | GO:0007004_telomere_maintenance_via_telomerase |  |  |  |  |  |  | GO:0032101_regulation_of_response_to_external_stimulus |  |  |
|  |  |  |  | GO:0006968_cellular_defense_response |  |  |  |  |  |  | GO:0031670_cellular_response_to_nutrient |  |  |
|  |  |  |  | GO:0006915_apoptosis |  |  |  |  |  |  | GO:0030817_regulation_of_cAMP_biosynthetic_process |  |  |
|  |  |  |  | GO:0006873_cellular_ion_homeostasis |  |  |  |  |  |  | GO:0030814_regulation_of_cAMP_metabolic_process |  |  |
|  |  |  |  | GO:0006790_sulfur_metabolic_process |  |  |  |  |  |  | GO:0030808_regulation_of_nucleotide_biosynthetic_process |  |  |
|  |  |  |  | GO:0006690_icosanoid_metabolic_process |  |  |  |  |  |  | GO:0030802_regulation_of_cyclic_nucleotide_biosynthetic_process |  |  |
|  |  |  |  | GO:0006446_regulation_of_translational_initiation |  |  |  |  |  |  | GO:0030799_regulation_of_cyclic_nucleotide_metabolic_process |  |  |
|  |  |  |  | GO:0006357_regulation_of_transcription_from_RNA_polymerase_II_promoter |  |  |  |  |  |  | GO:0030225_macrophage_differentiation |  |  |
|  |  |  |  | GO:0006325_chromatin_organization |  |  |  |  |  |  | GO:0030206_chondroitin_sulfate_biosynthetic_process |  |  |
|  |  |  |  | GO:0006278_RNA-dependent_DNA_replication |  |  |  |  |  |  | GO:0030204_chondroitin_sulfate_metabolic_process |  |  |
|  |  |  |  | GO:0003008_system_process |  |  |  |  |  |  | GO:0030182_neuron_differentiation |  |  |
|  |  |  |  | GO:0002889_regulation_of_immunoglobulin_mediated_immune_response |  |  |  |  |  |  | GO:0030005_cellular_di-__tri-valent_inorganic_cation_homeostasis |  |  |
|  |  |  |  | GO:0002822_regulation_of_adaptive_immune_response_based_on_somatic_recombination_of_immune_receptors_built_from_immunoglobulin_superfamily_domains |  |  |  |  |  |  | GO:0030003_cellular_cation_homeostasis |  |  |
|  |  |  |  | GO:0002819_regulation_of_adaptive_immune_response |  |  |  |  |  |  | GO:0022607_cellular_component_assembly |  |  |
|  |  |  |  | GO:0002762_negative_regulation_of_myeloid_leukocyte_differentiation |  |  |  |  |  |  | GO:0022603_regulation_of_anatomical_structure_morphogenesis |  |  |
|  |  |  |  | GO:0002715_regulation_of_natural_killer_cell_mediated_immunity |  |  |  |  |  |  | GO:0022411_cellular_component_disassembly |  |  |
|  |  |  |  | GO:0002712_regulation_of_B_cell_mediated_immunity |  |  |  |  |  |  | GO:0022008_neurogenesis |  |  |
|  |  |  |  | GO:0002706_regulation_of_lymphocyte_mediated_immunity |  |  |  |  |  |  | GO:0019932_second-messenger-mediated_signaling |  |  |
|  |  |  |  | GO:0002703_regulation_of_leukocyte_mediated_immunity |  |  |  |  |  |  | GO:0019752_carboxylic_acid_metabolic_process |  |  |
|  |  |  |  | GO:0002697_regulation_of_immune_effector_process |  |  |  |  |  |  | GO:0019725_cellular_homeostasis |  |  |
|  |  |  |  | GO:0002695_negative_regulation_of_leukocyte_activation |  |  |  |  |  |  | GO:0019395_fatty_acid_oxidation |  |  |
|  |  |  |  | GO:0002521_leukocyte_differentiation |  |  |  |  |  |  | GO:0019362_pyridine_nucleotide_metabolic_process |  |  |
|  |  |  |  | GO:0002460_adaptive_immune_response_based_on_somatic_recombination_of_immune_receptors_built_from_immunoglobulin_superfamily_domains |  |  |  |  |  |  | GO:0019321_pentose_metabolic_process |  |  |
|  |  |  |  | GO:0002449_lymphocyte_mediated_immunity |  |  |  |  |  |  | GO:0019229_regulation_of_vasoconstriction |  |  |
|  |  |  |  | GO:0002443_leukocyte_mediated_immunity |  |  |  |  |  |  | GO:0019217_regulation_of_fatty_acid_metabolic_process |  |  |
|  |  |  |  | GO:0002253_activation_of_immune_response |  |  |  |  |  |  | GO:0016568_chromatin_modification |  |  |
|  |  |  |  | GO:0002252_immune_effector_process |  |  |  |  |  |  | GO:0016197_endosome_transport |  |  |
|  |  |  |  | GO:0002250_adaptive_immune_response |  |  |  |  |  |  | GO:0016125_sterol_metabolic_process |  |  |
|  |  |  |  | GO:0002228_natural_killer_cell_mediated_immunity |  |  |  |  |  |  | GO:0016053_organic_acid_biosynthetic_process |  |  |
|  |  |  |  | GO:0001934_positive_regulation_of_protein_amino_acid_phosphorylation |  |  |  |  |  |  | GO:0015914_phospholipid_transport |  |  |
|  |  |  |  | GO:0001933_negative_regulation_of_protein_amino_acid_phosphorylation |  |  |  |  |  |  | GO:0010959_regulation_of_metal_ion_transport |  |  |
|  |  |  |  | GO:0001932_regulation_of_protein_amino_acid_phosphorylation |  |  |  |  |  |  | GO:0010926_anatomical_structure_formation |  |  |
|  |  |  |  | GO:0001910_regulation_of_leukocyte_mediated_cytotoxicity |  |  |  |  |  |  | GO:0010863_positive_regulation_of_phospholipase_C_activity |  |  |
|  |  |  |  | GO:0001909_leukocyte_mediated_cytotoxicity |  |  |  |  |  |  | GO:0010770_positive_regulation_of_cell_morphogenesis_involved_in_differentiation |  |  |
|  |  |  |  | GO:0001906_cell_killing |  |  |  |  |  |  | GO:0010718_positive_regulation_of_epithelial_to_mesenchymal_transition |  |  |
|  |  |  |  | GO:0001817_regulation_of_cytokine_production |  |  |  |  |  |  | GO:0010717_regulation_of_epithelial_to_mesenchymal_transition |  |  |
|  |  |  |  | GO:0001816_cytokine_production |  |  |  |  |  |  | GO:0010639_negative_regulation_of_organelle_organization |  |  |
|  |  |  |  | GO:0001672_regulation_of_chromatin_assembly_or_disassembly |  |  |  |  |  |  | GO:0010608_posttranscriptional_regulation_of_gene_expression |  |  |
|  |  |  |  | GO:0000279_M_phase |  |  |  |  |  |  | GO:0010522_regulation_of_calcium_ion_transport_into_cytosol |  |  |
|  |  |  |  | GO:0000075_cell_cycle_checkpoint |  |  |  |  |  |  | GO:0010518_positive_regulation_of_phospholipase_activity |  |  |
|  |  |  |  |  |  |  |  |  |  |  | GO:0010517_regulation_of_phospholipase_activity |  |  |
|  |  |  |  |  |  |  |  |  |  |  | GO:0009887_organ_morphogenesis |  |  |
|  |  |  |  |  |  |  |  |  |  |  | GO:0009820_alkaloid_metabolic_process |  |  |
|  |  |  |  |  |  |  |  |  |  |  | GO:0009792_embryonic_development_ending_in_birth_or_egg_hatching |  |  |
|  |  |  |  |  |  |  |  |  |  |  | GO:0009719_response_to_endogenous_stimulus |  |  |
|  |  |  |  |  |  |  |  |  |  |  | GO:0009653_anatomical_structure_morphogenesis |  |  |
|  |  |  |  |  |  |  |  |  |  |  | GO:0009615_response_to_virus |  |  |
|  |  |  |  |  |  |  |  |  |  |  | GO:0009607_response_to_biotic_stimulus |  |  |
|  |  |  |  |  |  |  |  |  |  |  | GO:0009395_phospholipid_catabolic_process |  |  |
|  |  |  |  |  |  |  |  |  |  |  | GO:0009308_amine_metabolic_process |  |  |
|  |  |  |  |  |  |  |  |  |  |  | GO:0009266_response_to_temperature_stimulus |  |  |
|  |  |  |  |  |  |  |  |  |  |  | GO:0009190_cyclic_nucleotide_biosynthetic_process |  |  |
|  |  |  |  |  |  |  |  |  |  |  | GO:0009187_cyclic_nucleotide_metabolic_process |  |  |
|  |  |  |  |  |  |  |  |  |  |  | GO:0009124_nucleoside_monophosphate_biosynthetic_process |  |  |
|  |  |  |  |  |  |  |  |  |  |  | GO:0009123_nucleoside_monophosphate_metabolic_process |  |  |
|  |  |  |  |  |  |  |  |  |  |  | GO:0009108_coenzyme_biosynthetic_process |  |  |
|  |  |  |  |  |  |  |  |  |  |  | GO:0009100_glycoprotein_metabolic_process |  |  |
|  |  |  |  |  |  |  |  |  |  |  | GO:0009062_fatty_acid_catabolic_process |  |  |
|  |  |  |  |  |  |  |  |  |  |  | GO:0008637_apoptotic_mitochondrial_changes |  |  |
|  |  |  |  |  |  |  |  |  |  |  | GO:0008544_epidermis_development |  |  |
|  |  |  |  |  |  |  |  |  |  |  | GO:0008284_positive_regulation_of_cell_proliferation |  |  |
|  |  |  |  |  |  |  |  |  |  |  | GO:0008203_cholesterol_metabolic_process |  |  |
|  |  |  |  |  |  |  |  |  |  |  | GO:0007588_excretion |  |  |
|  |  |  |  |  |  |  |  |  |  |  | GO:0007423_sensory_organ_development |  |  |
|  |  |  |  |  |  |  |  |  |  |  | GO:0007420_brain_development |  |  |
|  |  |  |  |  |  |  |  |  |  |  | GO:0007264_small_GTPase_mediated_signal_transduction |  |  |
|  |  |  |  |  |  |  |  |  |  |  | GO:0007214_gamma-aminobutyric_acid_signaling_pathway |  |  |
|  |  |  |  |  |  |  |  |  |  |  | GO:0007204_elevation_of_cytosolic_calcium_ion_concentration |  |  |
|  |  |  |  |  |  |  |  |  |  |  | GO:0007202_activation_of_phospholipase_C_activity |  |  |
|  |  |  |  |  |  |  |  |  |  |  | GO:0007187_G-protein_signaling__coupled_to_cyclic_nucleotide_second_messenger |  |  |
|  |  |  |  |  |  |  |  |  |  |  | GO:0007184_SMAD_protein_nuclear_translocation |  |  |
|  |  |  |  |  |  |  |  |  |  |  | GO:0007178_transmembrane_receptor_protein_serine_threonine_kinase_signaling_pathway |  |  |
|  |  |  |  |  |  |  |  |  |  |  | GO:0007127_meiosis_I |  |  |
|  |  |  |  |  |  |  |  |  |  |  | GO:0007126_meiosis |  |  |
|  |  |  |  |  |  |  |  |  |  |  | GO:0006986_response_to_unfolded_protein |  |  |
|  |  |  |  |  |  |  |  |  |  |  | GO:0006941_striated_muscle_contraction |  |  |
|  |  |  |  |  |  |  |  |  |  |  | GO:0006913_nucleocytoplasmic_transport |  |  |
|  |  |  |  |  |  |  |  |  |  |  | GO:0006879_cellular_iron_ion_homeostasis |  |  |
|  |  |  |  |  |  |  |  |  |  |  | GO:0006875_cellular_metal_ion_homeostasis |  |  |
|  |  |  |  |  |  |  |  |  |  |  | GO:0006874_cellular_calcium_ion_homeostasis |  |  |
|  |  |  |  |  |  |  |  |  |  |  | GO:0006816_calcium_ion_transport |  |  |
|  |  |  |  |  |  |  |  |  |  |  | GO:0006769_nicotinamide_metabolic_process |  |  |
|  |  |  |  |  |  |  |  |  |  |  | GO:0006767_water-soluble_vitamin_metabolic_process |  |  |
|  |  |  |  |  |  |  |  |  |  |  | GO:0006749_glutathione_metabolic_process |  |  |
|  |  |  |  |  |  |  |  |  |  |  | GO:0006740_NADPH_regeneration |  |  |
|  |  |  |  |  |  |  |  |  |  |  | GO:0006739_NADP_metabolic_process |  |  |
|  |  |  |  |  |  |  |  |  |  |  | GO:0006733_oxidoreduction_coenzyme_metabolic_process |  |  |
|  |  |  |  |  |  |  |  |  |  |  | GO:0006725_cellular_aromatic_compound_metabolic_process |  |  |
|  |  |  |  |  |  |  |  |  |  |  | GO:0006695_cholesterol_biosynthetic_process |  |  |
|  |  |  |  |  |  |  |  |  |  |  | GO:0006693_prostaglandin_metabolic_process |  |  |
|  |  |  |  |  |  |  |  |  |  |  | GO:0006692_prostanoid_metabolic_process |  |  |
|  |  |  |  |  |  |  |  |  |  |  | GO:0006635_fatty_acid_beta-oxidation |  |  |
|  |  |  |  |  |  |  |  |  |  |  | GO:0006633_fatty_acid_biosynthetic_process |  |  |
|  |  |  |  |  |  |  |  |  |  |  | GO:0006629_lipid_metabolic_process |  |  |
|  |  |  |  |  |  |  |  |  |  |  | GO:0006412_translation |  |  |
|  |  |  |  |  |  |  |  |  |  |  | GO:0006403_RNA_localization |  |  |
|  |  |  |  |  |  |  |  |  |  |  | GO:0006334_nucleosome_assembly |  |  |
|  |  |  |  |  |  |  |  |  |  |  | GO:0006333_chromatin_assembly_or_disassembly |  |  |
|  |  |  |  |  |  |  |  |  |  |  | GO:0006323_DNA_packaging |  |  |
|  |  |  |  |  |  |  |  |  |  |  | GO:0006171_cAMP_biosynthetic_process |  |  |
|  |  |  |  |  |  |  |  |  |  |  | GO:0006164_purine_nucleotide_biosynthetic_process |  |  |
|  |  |  |  |  |  |  |  |  |  |  | GO:0006140_regulation_of_nucleotide_metabolic_process |  |  |
|  |  |  |  |  |  |  |  |  |  |  | GO:0006119_oxidative_phosphorylation |  |  |
|  |  |  |  |  |  |  |  |  |  |  | GO:0006098_pentose-phosphate_shunt |  |  |
|  |  |  |  |  |  |  |  |  |  |  | GO:0006081_cellular_aldehyde_metabolic_process |  |  |
|  |  |  |  |  |  |  |  |  |  |  | GO:0006066_alcohol_metabolic_process |  |  |
|  |  |  |  |  |  |  |  |  |  |  | GO:0006006_glucose_metabolic_process |  |  |
|  |  |  |  |  |  |  |  |  |  |  | GO:0003018_vascular_process_in_circulatory_system |  |  |
|  |  |  |  |  |  |  |  |  |  |  | GO:0002831_regulation_of_response_to_biotic_stimulus |  |  |
|  |  |  |  |  |  |  |  |  |  |  | GO:0002768_immune_response-regulating_cell_surface_receptor_signaling_pathway |  |  |
|  |  |  |  |  |  |  |  |  |  |  | GO:0002763_positive_regulation_of_myeloid_leukocyte_differentiation |  |  |
|  |  |  |  |  |  |  |  |  |  |  | GO:0002761_regulation_of_myeloid_leukocyte_differentiation |  |  |
|  |  |  |  |  |  |  |  |  |  |  | GO:0002696_positive_regulation_of_leukocyte_activation |  |  |
|  |  |  |  |  |  |  |  |  |  |  | GO:0002683_negative_regulation_of_immune_system_process |  |  |
|  |  |  |  |  |  |  |  |  |  |  | GO:0002573_myeloid_leukocyte_differentiation |  |  |
|  |  |  |  |  |  |  |  |  |  |  | GO:0002429_immune_response-activating_cell_surface_receptor_signaling_pathway |  |  |
|  |  |  |  |  |  |  |  |  |  |  | GO:0002260_lymphocyte_homeostasis |  |  |
|  |  |  |  |  |  |  |  |  |  |  | GO:0001944_vasculature_development |  |  |
|  |  |  |  |  |  |  |  |  |  |  | GO:0001890_placenta_development |  |  |
|  |  |  |  |  |  |  |  |  |  |  | GO:0001836_release_of_cytochrome_c_from_mitochondria |  |  |
|  |  |  |  |  |  |  |  |  |  |  | GO:0001819_positive_regulation_of_cytokine_production |  |  |
|  |  |  |  |  |  |  |  |  |  |  | GO:0001776_leukocyte_homeostasis |  |  |
|  |  |  |  |  |  |  |  |  |  |  | GO:0001558_regulation_of_cell_growth |  |  |
|  |  |  |  |  |  |  |  |  |  |  | GO:0000902_cell_morphogenesis |  |  |
|  |  |  |  |  |  |  |  |  |  |  | GO:0000819_sister_chromatid_segregation |  |  |
|  |  |  |  |  |  |  |  |  |  |  | GO:0000226_microtubule_cytoskeleton_organization |  |  |
|  |  |  |  |  |  |  |  |  |  |  | GO:0000070_mitotic_sister_chromatid_segregation |  |  |
