# Supplementary material for: Functional Categories Associated with Clusters of Genes That Are Co-Expressed across the NCI-60 Cancer Cell Lines
Source: PLoS One. 2012 Jan 24;7(1):e30317. doi: 10.1371/journal.pone.0030317 (PMC3265467; doi:10.1371/journal.pone.0030317)
Supplement: Powerpoint S1 — Database tutorial. (PPT) [file pone.0030317.s009.ppt]

## Slide 1
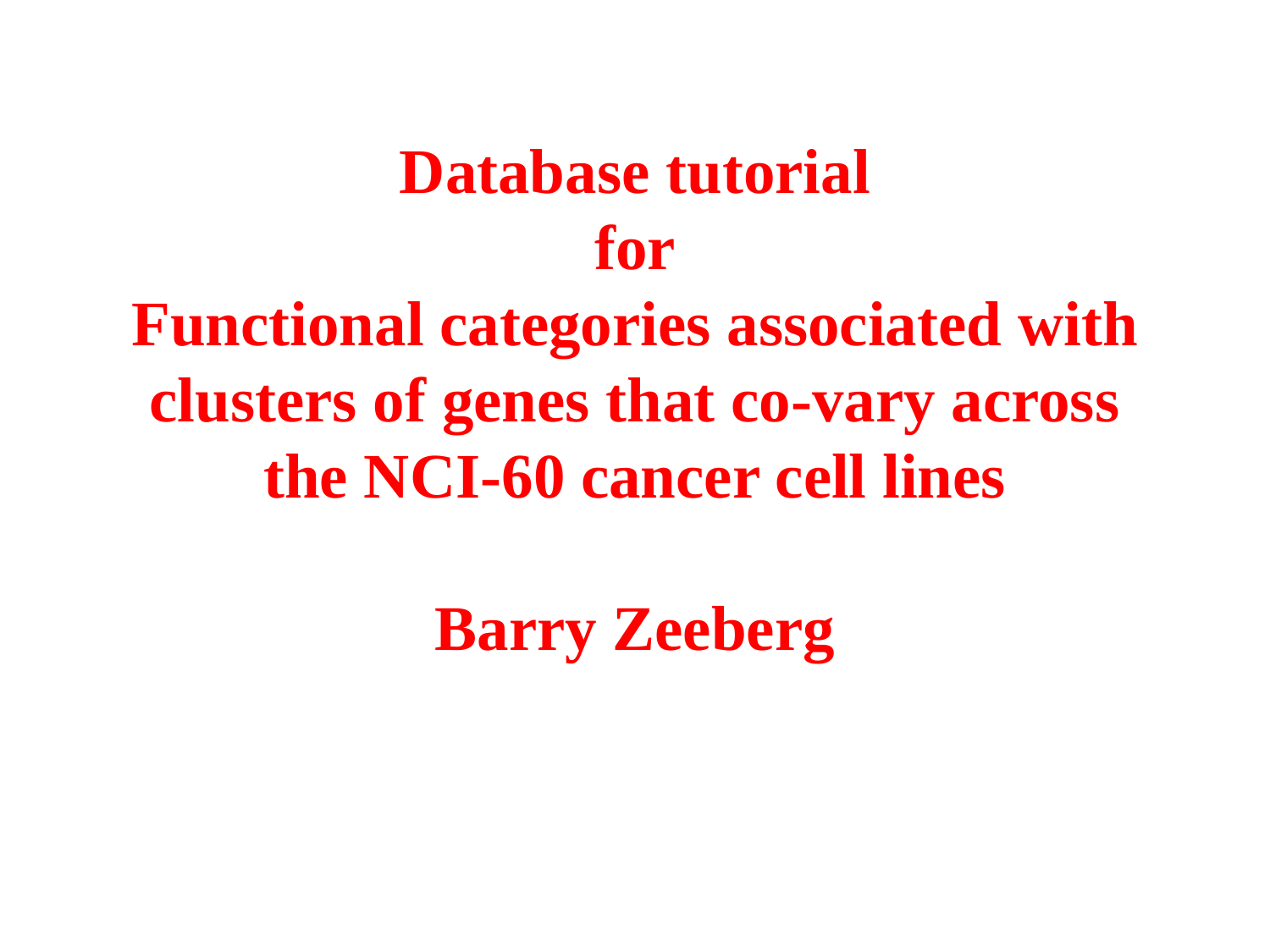

# Database tutorial for Functional categories associated with clusters of genes that co-vary across the NCI-60 cancer cell lines Barry Zeeberg

## Slide 2
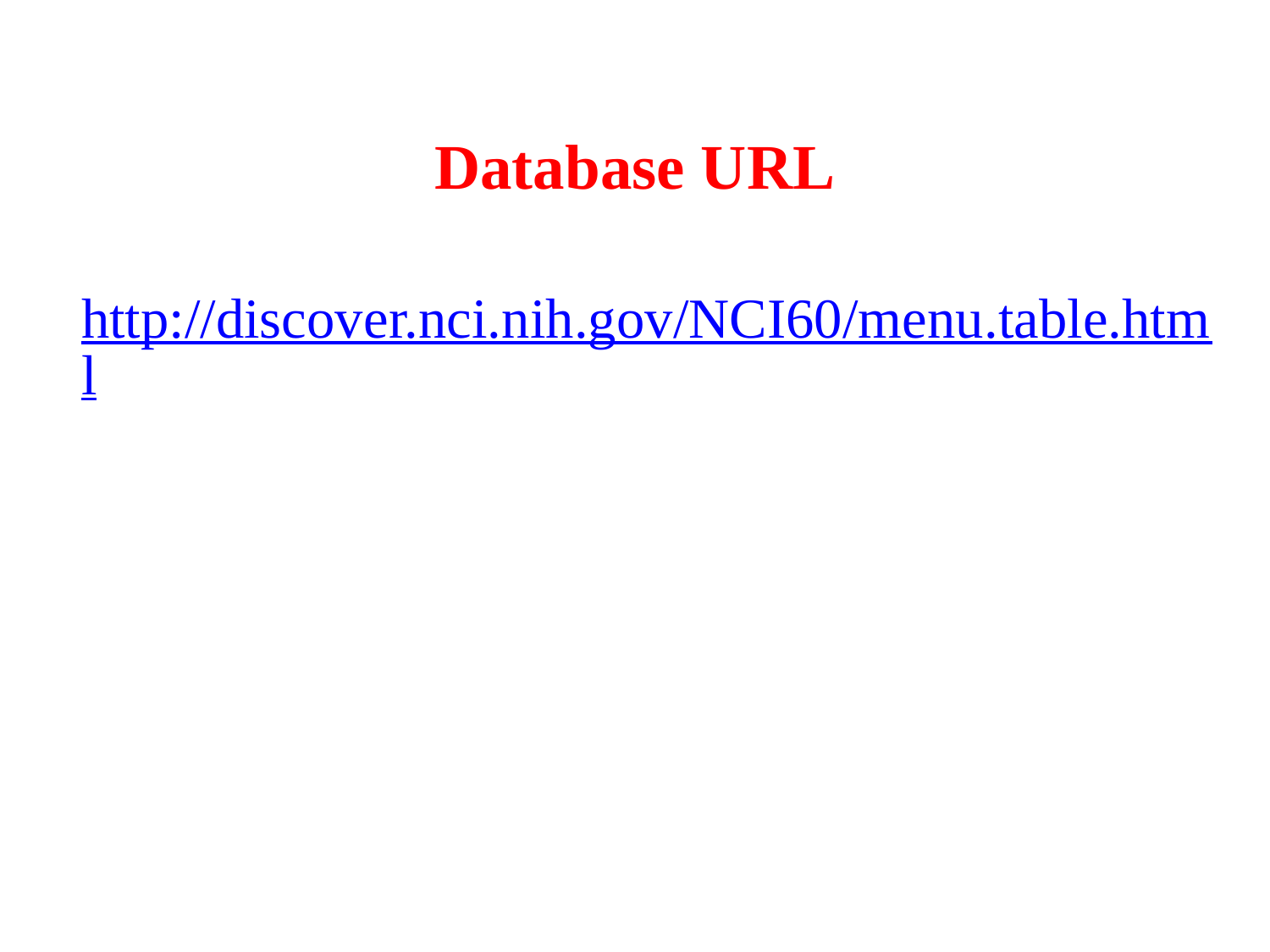

# Database URL
http://discover.nci.nih.gov/NCI60/menu.table.html

## Slide 3
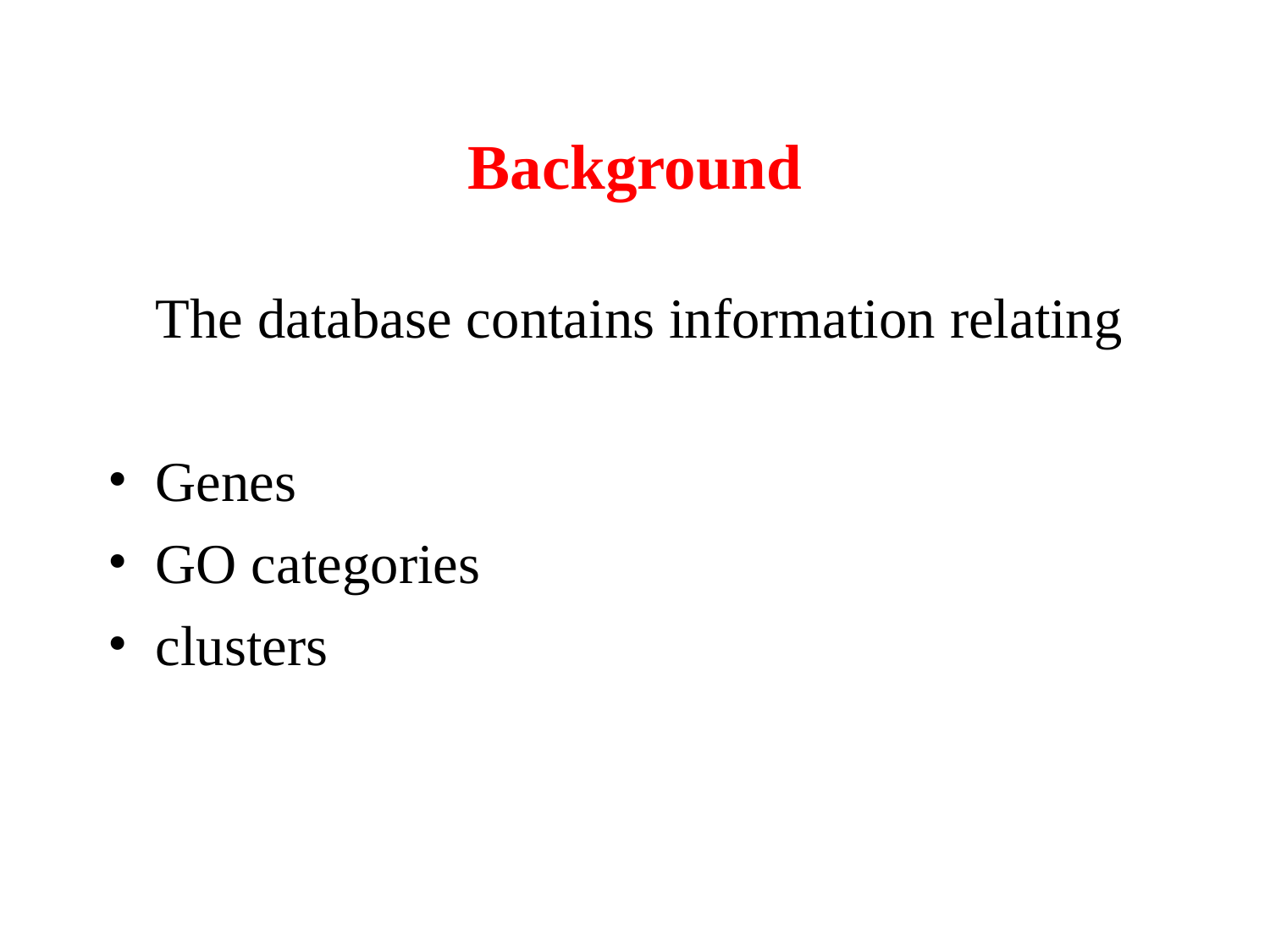

# Background
The database contains information relating
Genes
GO categories
clusters

## Slide 4
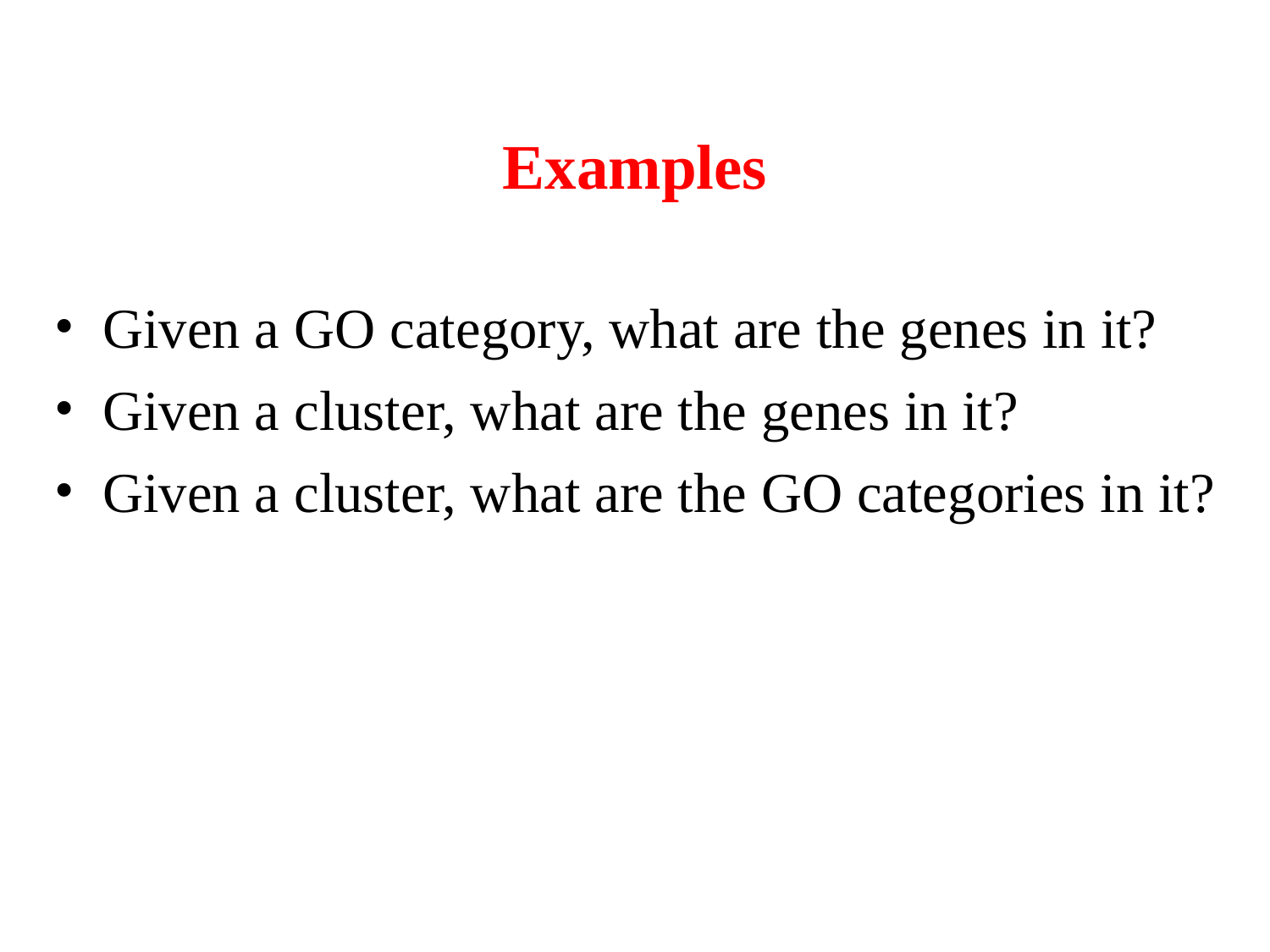

# Examples
Given a GO category, what are the genes in it?
Given a cluster, what are the genes in it?
Given a cluster, what are the GO categories in it?

## Slide 5
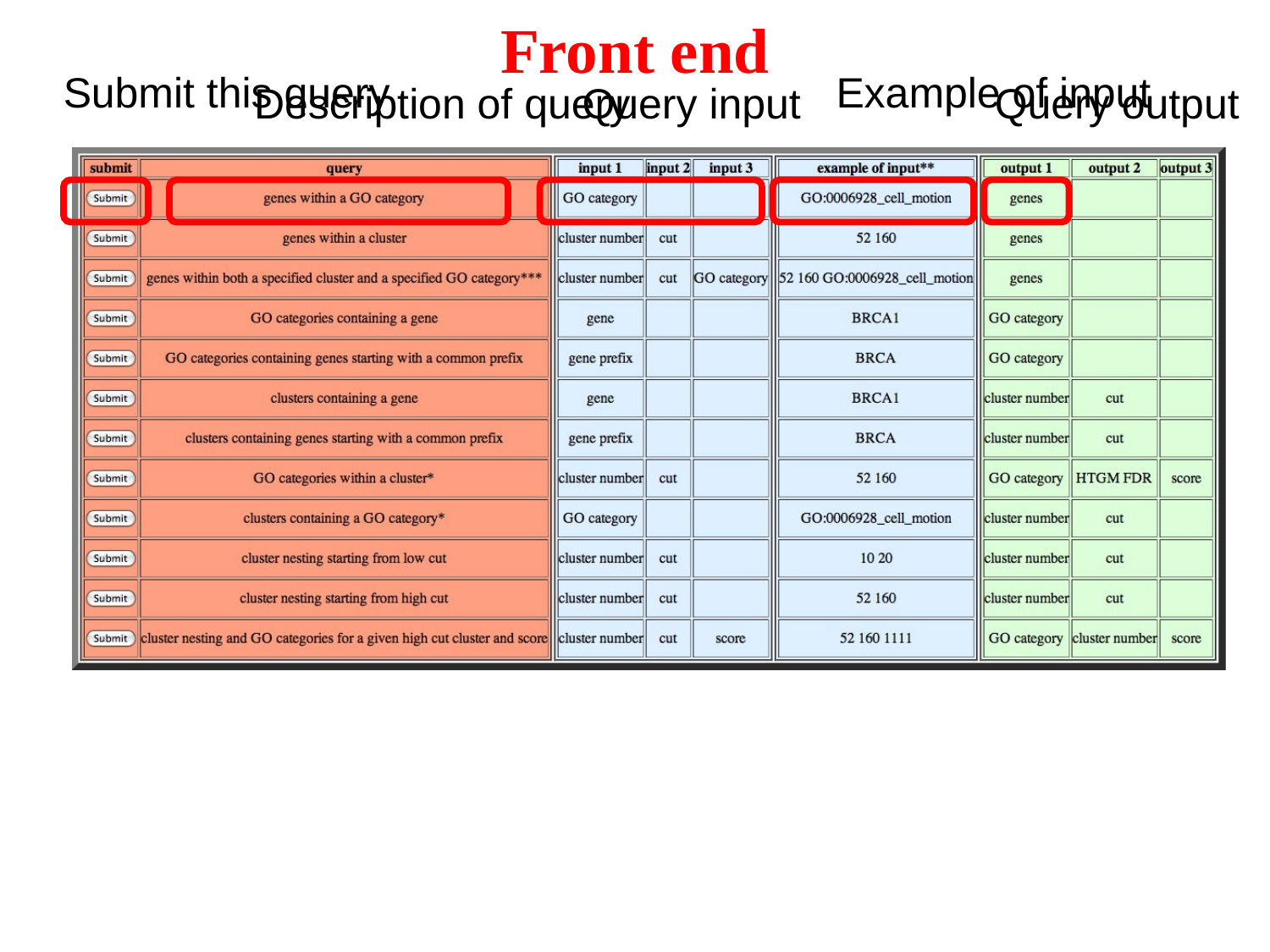

# Front end
Submit this query
Example of input
Description of query
Query input
Query output

## Slide 6
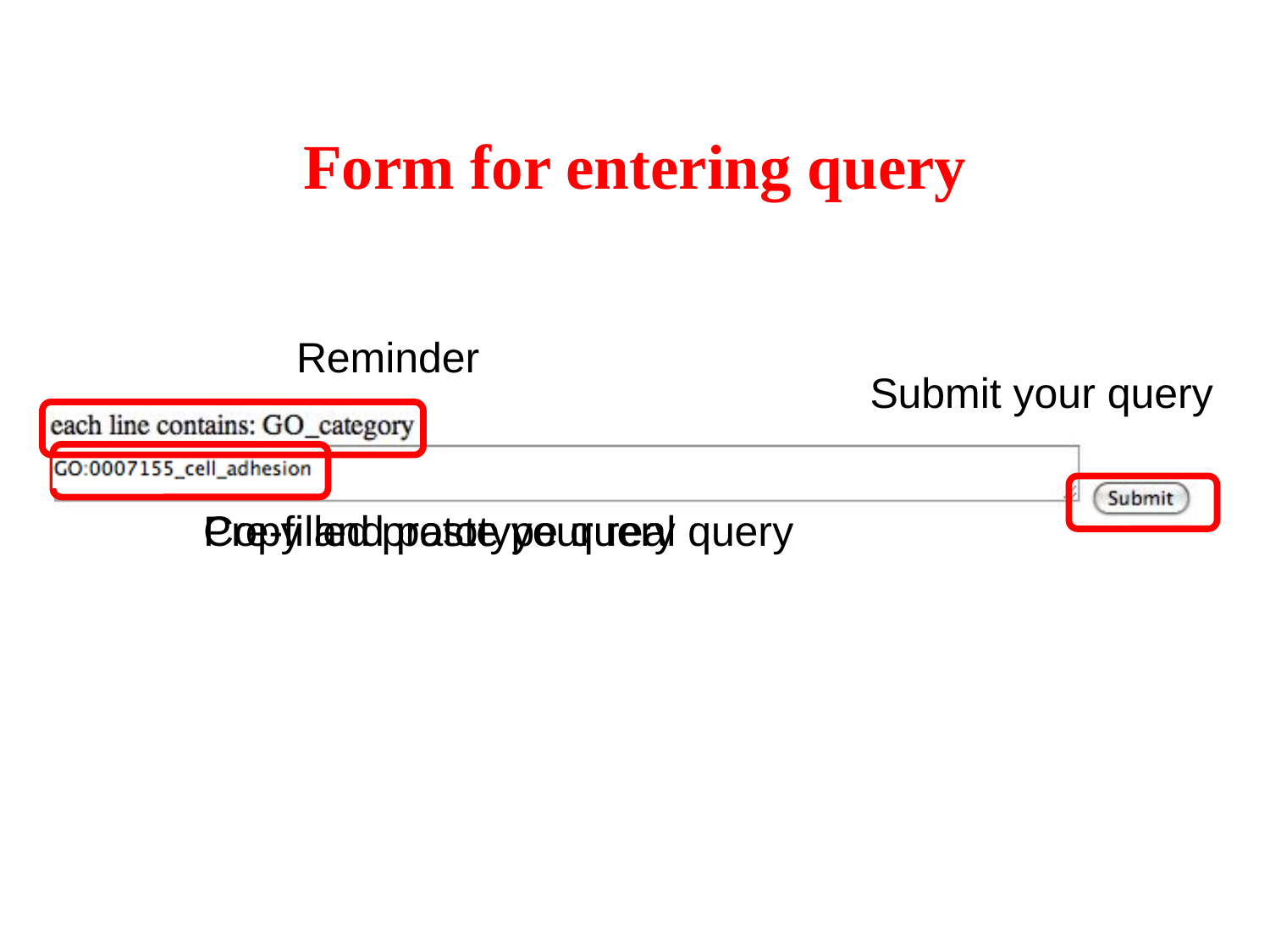

# Form for entering query
Reminder
Submit your query
Pre-filled prototype query
Copy and paste your real query

## Slide 7
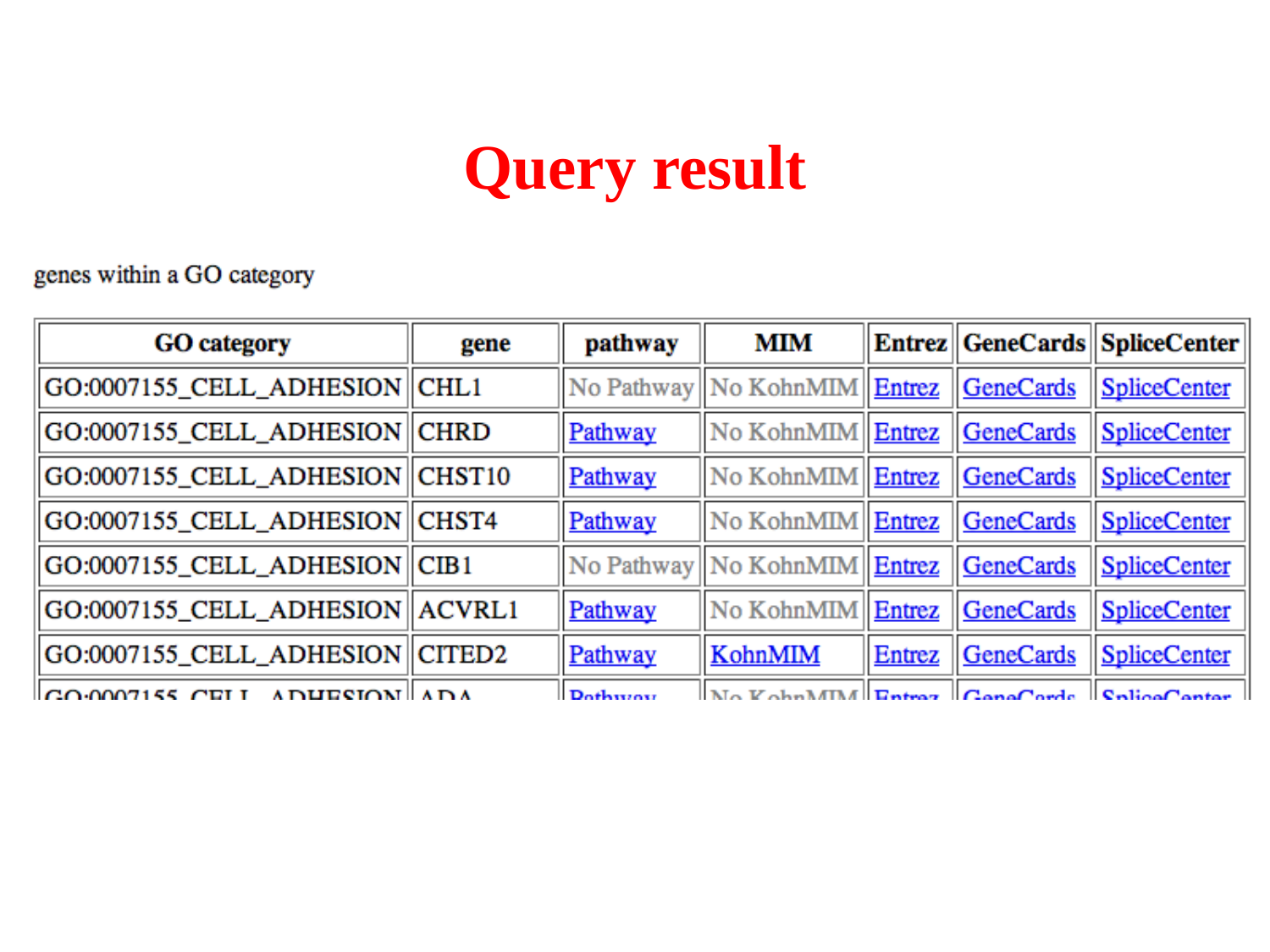

# Query result

## Slide 8
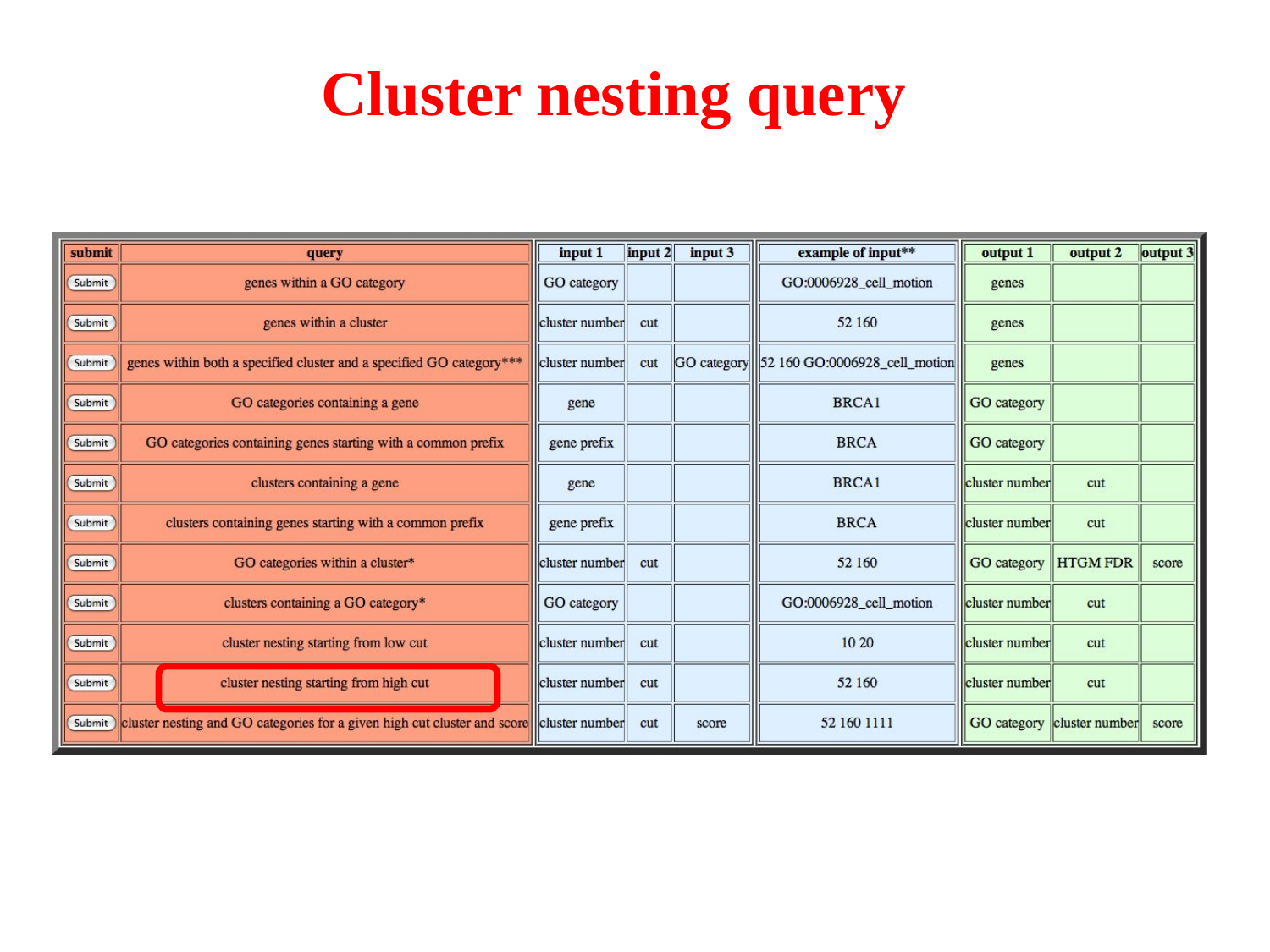

Cluster nesting query

## Slide 9
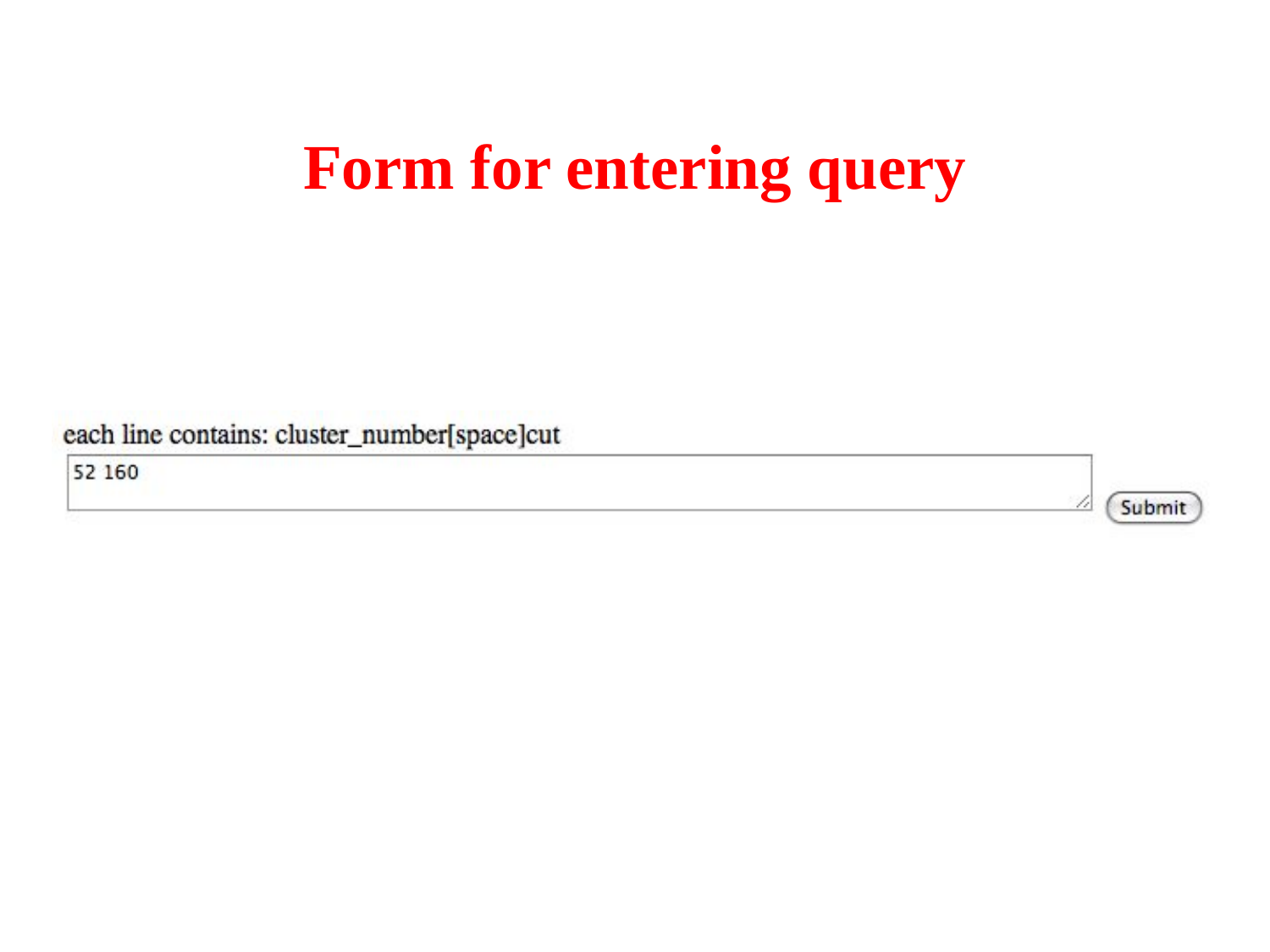

# Form for entering query

## Slide 10
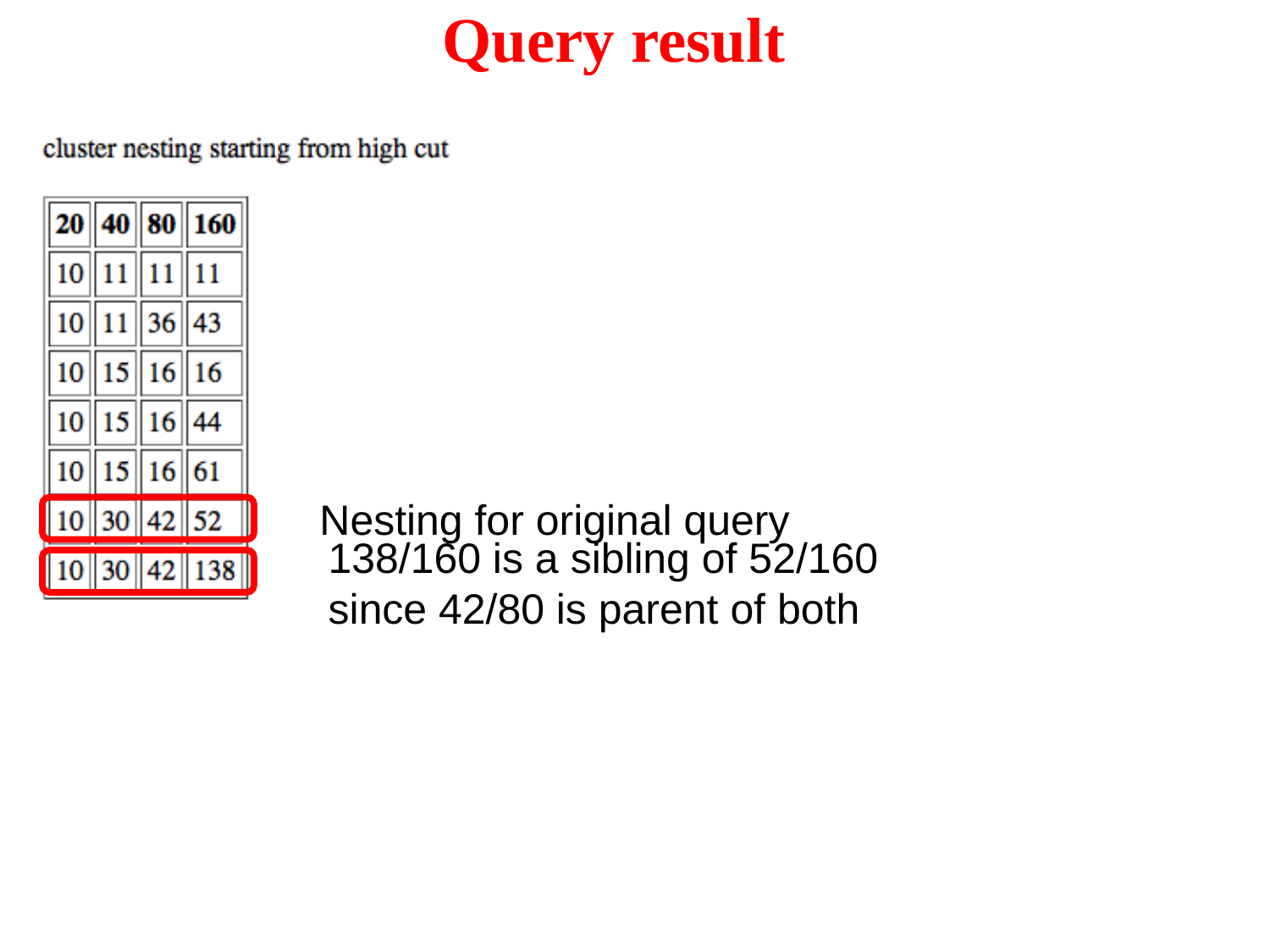

# Query result
Nesting for original query
138/160 is a sibling of 52/160
since 42/80 is parent of both

## Slide 11
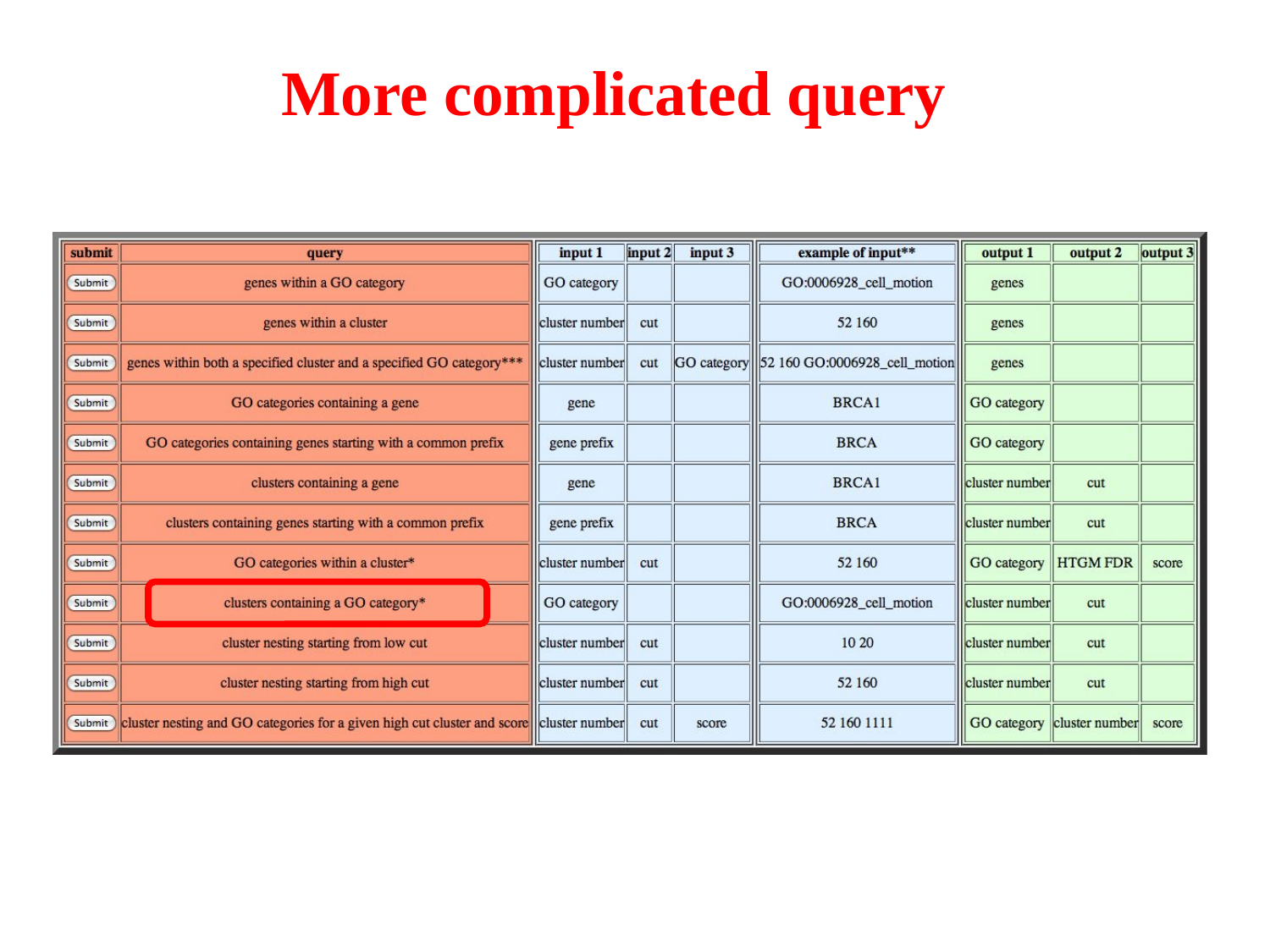

More complicated query

## Slide 12
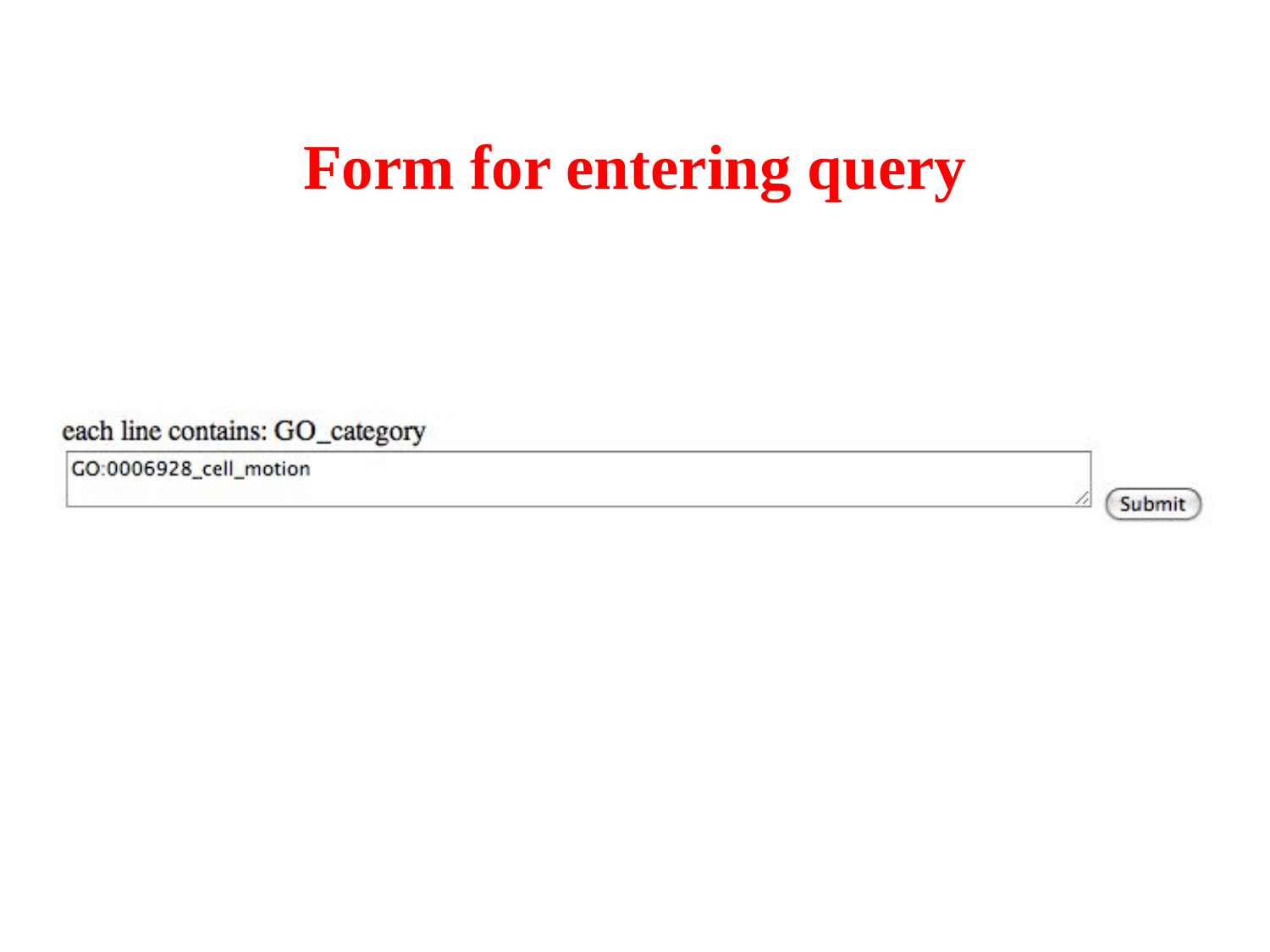

# Form for entering query

## Slide 13
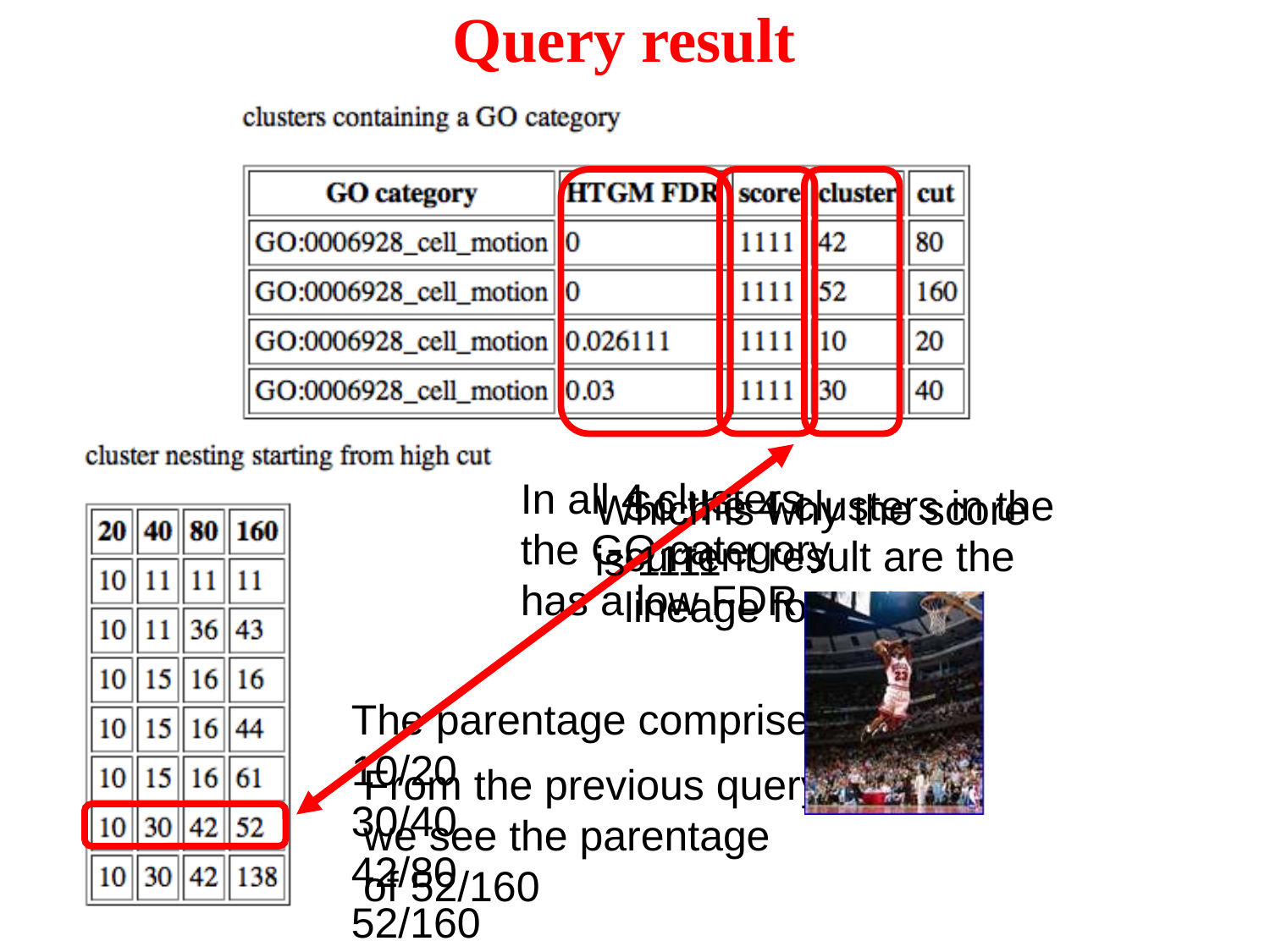

# Query result
In all 4 clusters
the GO category
has a low FDR
So the 4 clusters in the
current result are the
lineage for 52/160
Which is why the score
is 1111
The parentage comprises:
10/20
30/40
42/80
52/160
From the previous query,
we see the parentage
of 52/160
