# Supplementary figures and images for: Functional Categories Associated with Clusters of Genes That Are Co-Expressed across the NCI-60 Cancer Cell Lines
Source: PLoS One. 2012 Jan 24;7(1):e30317. doi: 10.1371/journal.pone.0030317 (PMC3265467; doi:10.1371/journal.pone.0030317)

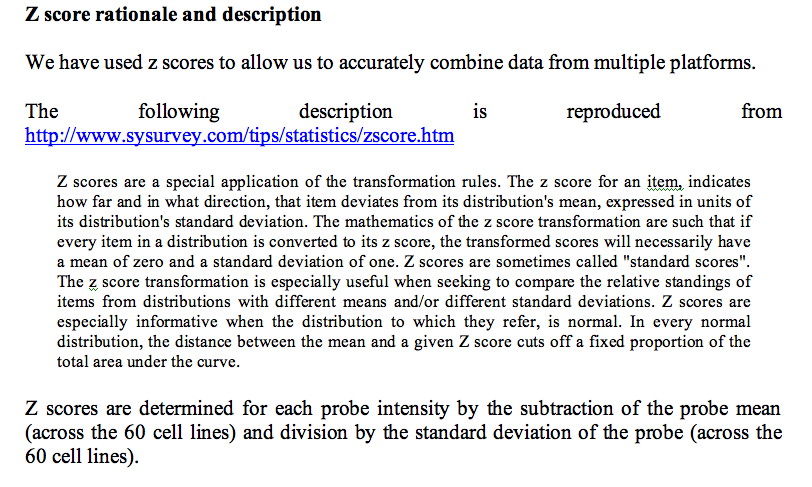

Supplement: Document S1 — Z-scores. (JPG) [file pone.0030317.s010.jpg]
