# Supplementary material for: Functional Categories Associated with Clusters of Genes That Are Co-Expressed across the NCI-60 Cancer Cell Lines
Source: PLoS One. 2012 Jan 24;7(1):e30317. doi: 10.1371/journal.pone.0030317 (PMC3265467; doi:10.1371/journal.pone.0030317)
Supplement: Figure S1 — CIM of categories versus cluster groups. (A) Each cluster group consists of a set of related clusters from the 20-, 40-, 80-, and 160-cut. The clusters are related by successive splittings from the 20-cut cluster. Clusters from a given cut are representd by a distinct color scale, as shown on the top of the figure. Robust categories and functionally-related adjacent regions are designated within a white rectangle. (B) Compacted version of CIM of categories categories versus cluster groups. Only those rows and columns containing robust categories were retained. The cluster number for the 160-cut is shown at the right of each encircled grouping. (PDF) [file pone.0030317.s001.pdf]

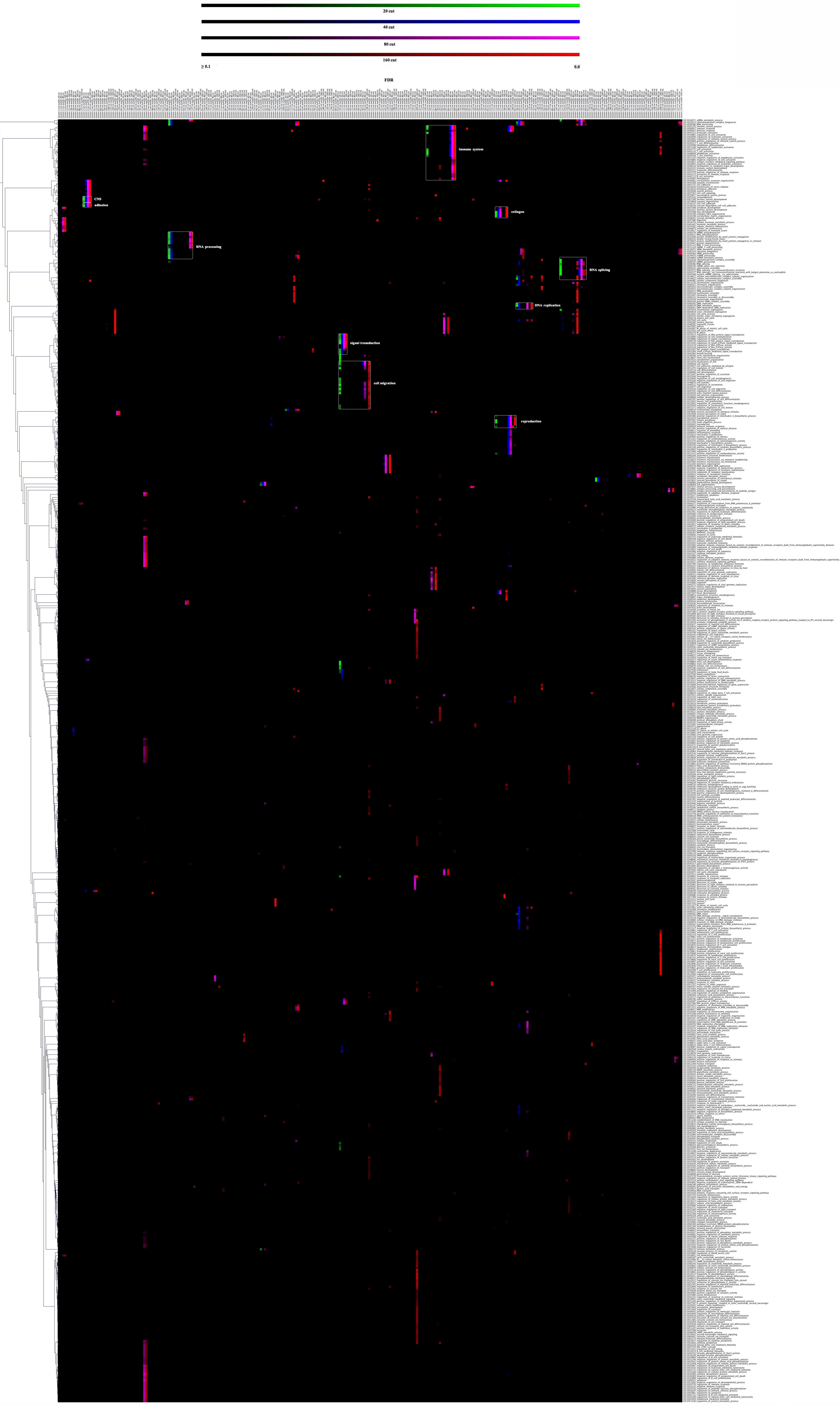

Figure S1A. CIM of categories versus cluster groups. Each cluster group consists of a set of related clusters from the 20, 40, 80, and 160 cut. The clusters are related by successive splittings from the 20 cut cluster. Clusters form a given cut are represented by a distinct color scale, as shown o the top of the figure. Robust categories and functionally-related adjacent regions are designated within a white rectangle.

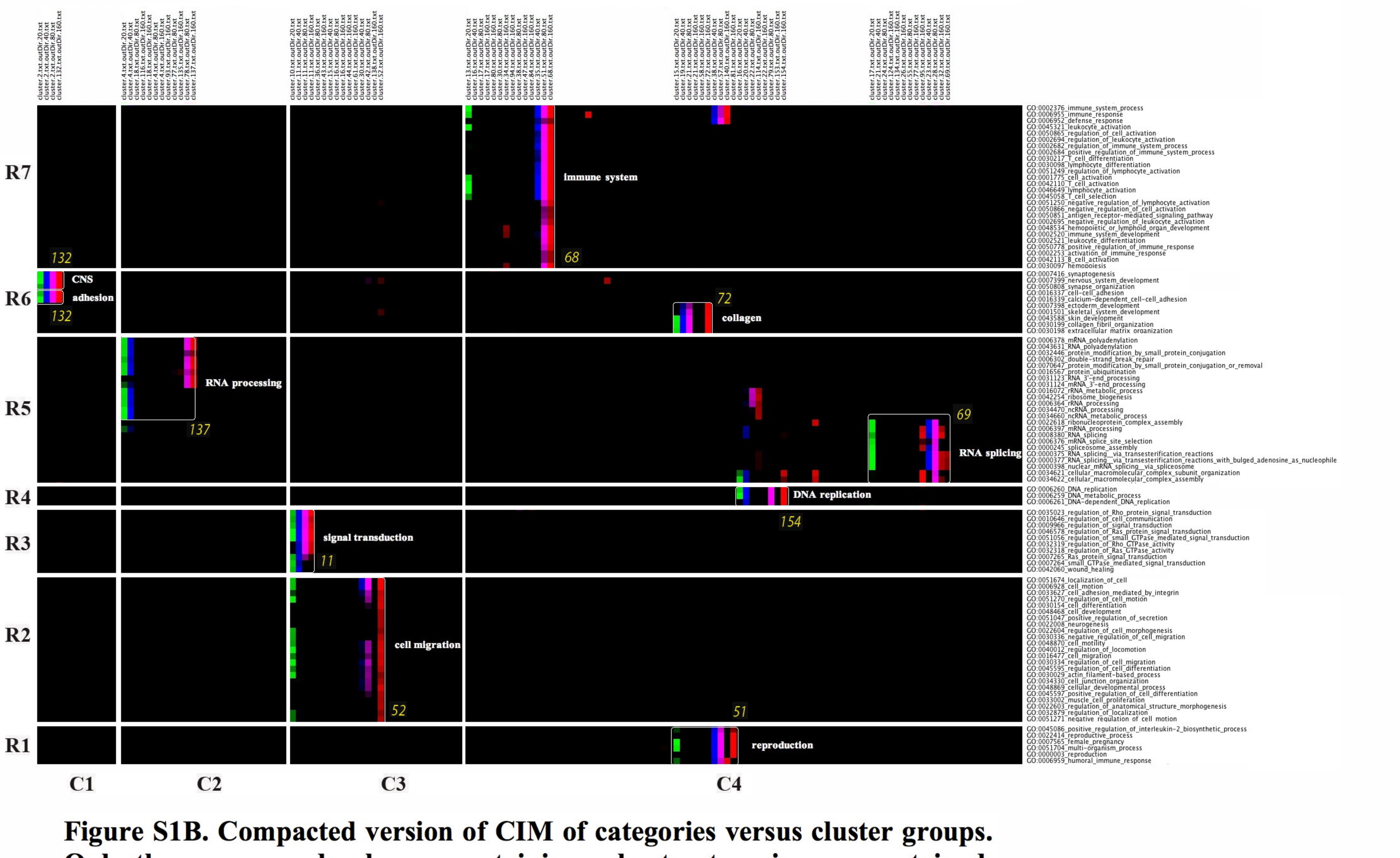

Figure S1B. Compacted version of CIM of categories versus cluster groups. Only those rows and columns containing robust categories were retained. The cluster number for the 160 cut is shown at the right of each encircled grouping.
